# Does Timing Matter? Exploring the Effects of Measurement Error on Models

**DOI:** 10.1007/s11538-026-01649-9

**Published:** 2026-05-19

**Authors:** Brock D. Sherlock, Marko A. A. Boon, Maria Vlasiou, Adelle C. F. Coster

**Affiliations:** 1 School of Mathematics & Statistics, University of New South Wales, Sydney, 2052 NSW Australia; 2 Department of Mathematics and Computer Science, Eindhoven University of Technology, P.O. Box 513, Eindhoven, 5600 MB The Netherlands; 3 Faculty of Electrical Engineering, Mathematics and Computer Science, University of Twente, P.O. Box 217, Enschede, 7500 AE The Netherlands

**Keywords:** Measurement Error, Error in Variables, Parameter inference, Data Fitting, Mathematical Modelling

## Abstract

Measurement error is an unavoidable feature of experimental data collection. It is common in mathematical biology to consider measurement error in the dependent variable. However, less attention has been given to errors in the independent variable. This work is focussed on the effects of independent variable measurement error in the biological sciences and the available statistical methods to account for these errors when performing parameter inference. Through a series of synthetic data studies, the effects of various error models are investigated, with a particular focus given to error in the time a measurement is taken. Across many scenarios, parameter inference proves robust to these errors, even without directly accounting for them. However, we find some systems, such as oscillating systems, are particularly susceptible to these errors and parameter estimates become biased. To aid researchers in the biological sciences, we review some statistical methods to correct for measurement error. We assess the applicability of these methods in a biological context by considering data availability and necessary assumptions for the methods. We find measurement error can have non-trivial and counter-intuitive effects on parameter inference and suggest assessing the available data should be an integral step in the modelling workflow. This allows researchers to identify when the integration of statistical methods to correct for measurement error are warranted.

## Introduction

Mathematical models are useful tools for interpreting and understanding a wide variety of data. Typically, data consists of measurements of a dependent variable *Y* which depends on some other value, the independent variable *X*. In the biological sciences, data is often noisy. This noise may be due to heterogeneity between individuals or measurement errors. Typically, a modeller seeks to fit a model, $Y=f(X)$, to this noisy data to infer system parameters or to understand the relationship between the independent and dependent variables. In this paper, we investigate the effects on inference when measurement errors in the independent variable are ignored (Section 3). For example, we find that oscillatory systems are particularly susceptible to measurement errors. We also review the available inference methods that account for these errors, highlighting the implications of different choices (Section 4). Our work provides modellers with the necessary information to make informed decisions when their data contains measurement errors.

The modelling literature has mainly focussed on errors (or noise) on the dependent variable. There are many methods for fitting data with errors on the dependent variable; see for example (Murphy et al. 2024; Qi and Baker 2025; Baltazar-Larios et al. 2025). However, in the modelling literature, less attention has been given to measurement error on the independent variable. That is, we do not observe a true value *X* but rather some observation *W* contaminated by an error *U*, such that $W = X + U$. Throughout this paper, we use *measurement error* to refer exclusively to errors in the independent variable. This is sometimes referred to as the *errors in variables* problem. We introduce common error models in Section 2.

Various studies find that ignoring measurement errors can lead to erroneous conclusions. For example, a bioassay experiment that investigated dose-response measures the concentration of herbicide absorbed by a plant with error (Rudemo et al. 1989). It was found that accounting for the measurement error significantly improved the model fit. A second set of examples are epidemiology studies that relate an outcome to the level of exposure to some chemical or radiation. In the first the exposure could not be measured directly and a surrogate measure was used (Tosteson et al. 1989; Pierce et al. 1992; Lyon et al. 2006) and in the second, an investigation with discrete variables, some instances had their diseased state misclassified (Carroll et al. 1993). In each case, it was found that neglecting components of measurement error could result in underestimating disease risk from exposure. Another example is the Framingham study (Kannel et al. 1986), a large cohort study for the development of coronary heart disease, in which predictors of coronary heart disease (such as cholesterol) were measured with error. Analyses found that the accuracy of the inferred probability of developing coronary heart disease given a predictor increased when measurement errors were accounted for (Buonaccorsi 2010; Carroll 2006). Epidemiological studies that require participants to respond to surveys, such as 24-hour diet recall, are often found to have a large measurement error. A lack of accounting for measurement errors may also be the cause of the discrepancy between these types of studies (Slimani et al. 2000; Beaton et al. 1979; Wu et al. 1988). Examples of measurement errors in ecological studies include the reliance on sampling the surrounding area for habitat variables or using the nearest monitoring station for weather variables (for example (Clark et al. 2001; Klaus and Buehler 2001)) – each can be a source of error, although no general impacts of the errors in this field were found. In general, however, the larger the spread of the true parameter values, the less the impact of measurement errors (Buonaccorsi 2010).

In the biological sciences, experiments can typically be categorised into two types: non-controlled and controlled experiments. In a non-controlled experiment, a random sample is taken from a population and both the independent and dependent variables are measured. On the other hand, a controlled experiment preassigns the independent variable, and observations of the dependent variable are made only for these preassigned values. Most of the previous examples given are non-controlled experiments. In this work, we are interested in experiments in which time is the independent variable with preset values, so we focus on controlled experiments.

In this study, we examine the effects of measurement error on parameter inference and model fitting when naive estimates (ignoring measurement error) are made. This is done through five numerical studies of common models found across the biological sciences in Section 3. We find that different systems and data structures are impacted by measurement errors to differing extents. For example, oscillatory systems are particularly susceptible to measurement errors. The estimation of the amplitude of the oscillations is found to be consistently underestimated. On the other hand, we find that there is a wide range of biological experiments in which measurement errors have minimal impacts. These are non-oscillating systems with multiple samples per time point and high heterogeneity between samples. In fact, the higher the heterogeneity, the lesser the impact of measurement errors.

The statistics and econometrics literature has widely studied methods for correcting for measurement error; see for example (Schennach 2016; Carroll 2006; Buonaccorsi 2010; Buzas et al. 2004; Fuller 1987; Wansbeek and Meijer 2003). We review various methods and techniques that account for measurement errors in Section 4, categorised by their assumptions and data requirements. The aim of this classification of the methods is to help those analysing data with measurement error identify which methods may be applicable and under what assumptions they are valid.

The potential benefits and drawbacks of applying these methods in the biological sciences are discussed in Section 5. We note that methods requiring additional data will have limited applicability in the biological sciences. In many scenarios, the required extra data is either not available due to experimental limitations or will come at the cost of requiring experimentalists to perform additional replicates. Methods that do not require additional data generally instead require assumptions on the distribution of the measurement error. The distribution of the error may not always be obvious and, when the variance of the errors is unknown, additional parameters then need to be inferred. This discussion of methods and their implications is intended to allow mathematical modellers in the biological sciences to make informed decisions for their particular setting.

The threefold impact of measurement errors is described in Carroll (2006). As the authors explain, measurement error causes a loss of statistical power when testing, bias in parameter estimation, and, for nonlinear models, a masking of features. In this work, we explore these effects in the context of controlled experiments subject to errors in the preset independent variable values, using case studies based on common experimental setups in the biological sciences. Our focus is on time-series studies, where system measurements are taken at predetermined time points. Repeat measurements at a time point are independent of one another, such as by being measurements taken in different wells of a plate or of different tumours. We find that the heterogeneity inherent in biological systems can diminish some of the effects of measurement error on parameter inference. In this paper, our aim is to raise awareness among biological researchers of the potential effects of measurement error in their data, and to present available correction methods applicable across different scenarios.

## Error Models

In this section, we introduce the notation and terminology used throughout this paper. We distinguish between the true independent variable measurement *X* and its observed counterpart *W*. We then introduce the two types of measurement error used in this paper, Berkson errors and classical errors.

***Notation*** A list of parameters and their definitions is given in Table 1. Throughout this paper, we use $Y_{ij}$ to denote the dependent random variable and $X_{ij}$ to denote the independent random variable(s) for the *i*th subject at the *j*th observation. When *X* cannot be observed directly or is measured with error, we use $W_{ij}$ to denote the observed value. When only considering paired observations, i.e., one dependent *Y* for a single independent variable *X*, we drop the subscripts to enhance readability. Capitalised variables (e.g. *Y*) refer to a random variable and lowercase (*y*) refers to a realised (or model-determined) value. For *X* and *Y* random variables, we denote by $\phi _X$ the probability density function for *X* and by $\phi _{X\vert Y}$ the conditional density of *X* given *Y*.

**Table 1:** Definitions of parameters

Parameter	Definition
*X*	True independent variable – measured without error
*Y*	True dependent variable
*U*	Measurement error in independent variable
*W*	Observed independent variable
*V*	Measurement error in dependent variable
*D*	Observed dependent variable
$\mu _K$	Mean for random variable *K*
$\sigma _K$	Standard deviation for random variable *K*
$\epsilon$	Error in the equation – an error term that distributes *Y* observations about the model equation
$\mathbb {E}[X]$	The expected value of a random variable *X*
$\mathbb {P}(x)$	Probability that $X=x$
$\theta$	Parameters to be estimated
$\hat{\theta }$	The estimated values of $\theta$
$\mathcal {L}(\theta \vert y)$	The likelihood function – the likelihood of parameters $\theta$ given the data *y*.
$\ell (\theta \vert y)$	The log-likelihood function, i.e., $\log (\mathcal {L}(\theta \vert y)$
$y(\cdot )$	A model for the data
$\phi _K$	The probability density function for random variable *K*

Capitalised values denote a random variable and the lowercase equivalent is a realisation of the random variable

***Types of Measurement Error*** In this paper, we consider two types of measurement error: Berkson errors (Berkson 1950) and classical (non-Berkson) errors. Systems with Berkson errors have the true values distributed around the observed values, $X_{ij} = W_{ij} + U_{ij}$, whereas systems with classical errors have the observed values distributed around the true values, $W_{ij} = X_{ij} + U_{ij}$. That is, for the classical errors the conditional distribution of the observed value *W* given the true value *X* is modelled, and for Berkson errors, the conditional distribution for *X* given *W* is modelled.

Berkson errors are typical when a measurement is assigned such as in a controlled experiment, for example when pre-assignments are made of a concentration of reagent to be applied or the time a measurement is to be taken. Berkson errors can also occur in non-controlled experiments such as if all individuals in a group are assigned an exposure to asbestos based on the years they have worked at a site. However, each individual has almost surely worked a different number of days over the years, worked in different locations on site, and had different work patterns resulting in different exposures (which would be distributed about the assigned value) (Carroll 2006). On the other hand, classical errors typically arise when an individual’s true value is measured with random noise, so that the observed value fluctuates around the true (unobserved) value. For example, repeated blood pressure measurements taken from a single participant will vary due to instrument precision, operator technique, and short-term physiological changes. Similarly, responses on a dietary recall survey may deviate from actual intake because of imperfect memory or uncertainties in estimating portion sizes. Some areas of the literature use the term classical error to describe the situation that the measurement error *U* is independent of the true value *X* and $\mathbb {E}[U]=0$ (the expected value of the error is zero). To avoid confusion, we will use the term *unbiased error* when the expected value of the error is zero, and *biased* when the expected value is non-zero.

Measurement errors can be further classified as differential or non-differential. A measurement error is said to be *non-differential* (with respect to *Y*) if the distribution of the observation given the independent and dependent variable $W \vert (X=x,Y=y)$ is equal to the distribution of the observation given only the independent model variable $W \vert (X=x)$. A measurement error model is said to be *differential* if the distribution of $W \vert (X=x,Y=y)$ changes with *y* (Buonaccorsi 2010; Fosgate 2006). Typically, *Y* is the dependent variable but this is not always the case.

In general, most scenarios will have non-differential error. However, there are two scenarios more likely to have differential error. The first is case-control or choice-based sampling studies. In these studies, the response is obtained first and the independent variables by follow-up. For example, evidence was found for differential errors in a study relating cervical cancer rates to exposure to herpes simplex virus type-2 (HSV-2) (Carroll et al. 1993). The true exposure to HSV-2 was measured with a refined western blot procedure, *X*, and a less accurate western blot procedure, *W*. The dependent variable *Y* is the indicator of cervical cancer. It was found that the probability of using the less accurate western blot procedure to measure exposure to HSV-2 resulted in a lower rate of misclassifying the presence of HSV-2 if the subject also had cervical cancer. That is, the probability of the less accurate measurement of HSV-2, *W*, being the true exposure to HSV-2, *X*, depends on the presence of cervical cancer, *Y*, i.e.,

$$\begin{aligned} \mathbb {P}(X=W \vert X=x,Y=0) \ne \mathbb {P}(X=W \vert X=x,Y=1). \end{aligned}$$

This suggests differential errors. It is important to note that these experiments allow the collection of measurements for the independent variable only after a diagnosis has been obtained. A second scenario for differential errors is when the observed independent variable *W* is not a mismeasured true value *X*, but instead a separate proxy for *X*. An example of this is a study that estimated coronary heart disease, *Y*, using low density lipoprotein cholesterol level (LDL) as *X* (Satten and Kupper 1993). The authors investigated using total cholesterol as a surrogate measure, *W*, of LDL. Both *X* and *W* are available in the data. Their analysis found a relationship between *W* and *Y*, even after correcting for *X*. In this study, we focus on non-differential errors as this is the natural setting for mismeasured time variables in controlled experiments. For a treatment of differential errors, see (Buonaccorsi 2010; Carroll et al. 1993; Carroll 2006).

## Naive Inference

In this section, we show the effects of ignoring measurement errors for parameter estimation. We demonstrate the effects (or lack thereof) of using naive inference in some common biological systems. Here, ‘naive inference’ refers to the use of models and inference techniques that do not explicitly consider measurement error on the independent variable. We start by reviewing several naive inference techniques, namely least squares, likelihood, and Bayesian methods – in Section 4 we will see alternative formulations for the likelihood that account for measurement error. We then provide an overview of some of the known effects of measurement error. Using five examples we illustrate scenarios in which measurement error either substantially alters the inference or has negligible impact.

### Parameter Inference

The aim of parameter inference is to find a set of parameters $\theta$ for a given model $y(\theta ,x)$ that best describes a given dataset. Our dataset consists of paired observations $(x_i,y_i)$ for $i=1,\dots ,N$ observations. The $x_i$ do not need to be distinct. That is, there can be tied or repeated observations. We do not consider model misspecification in this study and instead assume the modeller chooses the correct model to describe the data. That is, $y(\theta ,x)$ describes the true relationship of the data free of error. Here we assume the observations have an error on the dependent variable and the inference methods in this section consider the effects of only that error.

***Least Squares*** The least squares method estimates the best parameters $\theta$ for a model $y(\theta ,x)$ by solving the minimisation

1 $$\begin{aligned} \min _\theta \sum _{i=1}^N \left[ y_i - y(\theta ,x_i)\right] ^2, \end{aligned}$$

recalling $y_i$ is the observed response and $y(\theta ,x_i)$ is the model output at $x_i$.

A benefit of using least squares estimation is that no assumptions about distributions of the data or the errors are required. It provides a good basis for model comparisons when seeking qualitative agreement between the model and the data. However, the method is sensitive to outliers (Yu and Yao 2017) and does require assumptions about the distribution of the errors to construct confidence intervals.

***Likelihood Methods*** The aim of likelihood methods is to find the set of model parameters, $\hat{\theta }$, that maximise the likelihood function, $\mathcal {L}$, given the data, *y*, that is,

$$\begin{aligned} \hat{\theta } = \arg \,\!\max _\theta \mathcal {L}(\theta \vert y). \end{aligned}$$

The $\hat{\theta }$ that maximises the likelihood is known as the maximum likelihood estimator (MLE). The likelihood that the observations come from a system with true parameters $\theta$ is the probability of the observations given the parameters,

2 $$\begin{aligned} \mathcal {L}(\theta \vert y) = \mathbb {P}(y \vert \theta ). \end{aligned}$$

If we assume the data points are independent and identically distributed (iid), then the probability of observing the entire dataset, $\{y_i\}_{i=1,\ldots ,N}$, is given by the product of the probabilities of seeing each data point. That is,

3 $$\begin{aligned} \mathcal {L}(\theta \vert y) = \prod _{i=1}^N \phi _{Y \vert X}(y_i;x_i,\theta ). \end{aligned}$$

In the biological sciences, it is common to want to fit a mechanistic model $y(\theta ,x)$. In this case, the likelihood is a function of the model – how likely are the observations given the model and the model parameters, or

$$\begin{aligned} \mathcal {L}(\theta \vert y) = \prod _{i=1}^N \phi _{Y \vert X}(y_i;y(\theta ,x_i),\theta ), \end{aligned}$$

where $y(\theta ,x)$ is the model for the data (usually some deterministic function of both the parameters to be estimated and the dependent variable), and $y_i$ is the observed data (from the random variable *Y*) . The form of $\phi _{Y \vert X}(y_i;y(\theta ,x_i),\theta )$, for biological experiments, is usually the expected distribution of data about the deterministic model output. See Murphy et al. (2024) for an overview of common models for the density in the biological sciences.

It is common to work with the log-likelihood function in numerical implementations to avoid underflow. The log-likelihood function is given by

4 $$\begin{aligned} \ell (\theta \vert y) = \sum _{i=1}^N \log [\phi _{Y\vert X}(y_i; y(\theta ,x_i),\theta )]. \end{aligned}$$

Maximising the logarithm also maximises the likelihood. So, the MLE is then given by the $\theta$ that maximises Equation (4).

If the measurement errors of the independent variable are assumed to be additive, independent, and normally distributed about a deterministic model output with constant variance, $\sigma ^2$, i.e.,

$$\begin{aligned} y_i = y(\theta ,x_i) + \epsilon _i, \end{aligned}$$

with $\epsilon _i \sim \mathcal {N}(0,\sigma ^2)$ then the MLE is equivalent to the least squares estimate of the parameters. In the equivalent likelihood and log-likelihood functions $\phi$ is the probability density function for the normal distribution with mean $y(\theta ,x_i)$ and variance $\sigma ^2$, i.e., the probability of seeing the measurement $y_i$ at $x_i$ with parameters $\theta$. Rather than maximising the log-likelihood function, many contexts use the negative log-likelihood, which in this case becomes

5 $$\begin{aligned} -\log (\ell (\theta \vert y)) = \sum _{i=1}^N \frac{[y_i - y(\theta ,x_i)]^2}{2\sigma _i^2} + \frac{1}{2}\log \left( 2\pi \sigma _i^2\right) . \end{aligned}$$

As this is the negative log-likelihood, the $\theta$ that minimises Equation (5) will maximise the likelihood. As the second term does not depend on the parameters, we only need to minimise the first term,

$$\begin{aligned} \min _\theta \sum _{i=1}^N \frac{\left[ y_i - y(\theta ,x_i)\right] ^2}{2\sigma _i^2}. \end{aligned}$$

With the assumption that the variances, $\sigma _i^2$, are constant across the dataset this minimisation is identical to the least squares estimate given in Equation (1).

There exists a vast literature outlining methods for assessing the quality of the inference. For information regarding profile likelihoods, assessing the identifiability of parameters, and construction of confidence intervals see for instance (Simpson and Maclaren 2023; Hass et al. 2016; Kreutz et al. 2013). An assessment of methods of uncertainty quantification and guidelines to choosing the correct method for a given problem is given in Villaverde et al. (2023).

Advantages of likelihood methods include their applicability to a wide range of models and error distributions, and the ability to focus on parameters of inference by optimising out nuisance parameters (Simpson and Baker 2025). The downside of likelihood-based methods is the requirement of specifying the distribution of the data. Model misspecification (choosing the wrong distribution) can produce misleading results including inconsistent estimators and underestimation of the uncertainty in estimates (White 1982). There can also be challenges evaluating the likelihood function. For many scenarios in the biological sciences, the likelihood function is not tractable (Warne et al. 2024).

***Bayesian Inference*** Unlike the likelihood-based methods above, which are informed only by the data, Bayesian methods incorporate prior knowledge of the parameters. This prior knowledge may come from the literature, where previous fitting of similar data has been performed, or from expert knowledge on what parameter values would be considered reasonable.

Using the usual Bayesian framework, we approximate the posterior distribution of the model parameters, $\theta$, using the prior distribution, $\pi (x)$, and the likelihood function for the observed data, $\mathcal {L}(y\vert \theta )$, such that

$$\begin{aligned} \pi (\theta \vert y) \propto \mathcal {L}(y \vert \theta ) \pi (\theta ). \end{aligned}$$

There is debate within the literature as to whether one should use *non-informative* or *weakly informative priors* – which often yield similar results to frequentist-based analyses – or to use more informative priors that potentially over influence model results, particularly for analyses using small datasets (Konold et al. 2025). We direct the reader to Gelman et al. (2017); Van De Schoot et al. (2021); Lemoine (2019) for information regarding the selection of priors.

The posterior distributions often cannot be calculated analytically. Instead, the posteriors are approximated by sampling algorithms such as the Metropolis-Hastings algorithm (Metropolis et al. 1953; Hastings 1970), Gibbs sampling (Geman and Geman 1993; Gelfand and Smith 1990), and sequential Monte Carlo (SMC) (Chopin 2002; Del Moral et al. 2006; Bon et al. 2021; South et al. 2019).

Advantages of a Bayesian approach include statistical interpretations of the results via the posterior distributions (the interpretation of results is different for MLE) and the ability to incorporate prior knowledge of the system via the prior distributions. However, a subjective prior can influence results, particularly for small datasets. Another disadvantage is that estimating the posterior is often more computationally expensive than an equivalent MLE method due to sampling, especially for high-dimensional problems (Gelman et al. 2013).

### Effects of Measurement Errors on Naive Models

In this section, we explore the effects of ignoring (independent variable) measurement error on parameter inference. We highlight some results from the literature and demonstrate the effects for some specific systems with synthetic data studies.

The effects of measurement error for linear models are supported by a robust theoretical underpinning; see for example (Buonaccorsi 2010; Carroll 2006; Buzas et al. 2004; Meijer et al. 2021). We demonstrate with synthetic data the effects on the estimation of the slope parameter when data contains measurement error.

The effects of measurement error on nonlinear models are not as well understood theoretically. The effects are largely case-dependent – the effects depend on the model and the type of error. The effects of measurement errors are discussed in Carroll (2006) for a wide variety of models, and in Chen et al. (2011); Schennach (2016) with a focus on econometrics. Each reference attests that, unlike linear models, we cannot make general qualitative statements about the effects.

Synthetic data studies are used throughout the section to demonstrate the potential effects of measurement error for a range of models and error distributions. We focus on measurement error applied to time as the independent variable. Mismeasured variables are typical in longitudinal data for various reasons, such as equipment accuracy or human error. In some scenarios, protocol times are recorded instead of actual sampling times (Wang and Davidian 1996). We perform naive inference, that is, inference without explicitly accounting for measurement errors, for a linear system, an oscillating system, and three different growth models for a variety of measurement errors.

Unless otherwise specified, the scenarios investigated below were fitted using nonlinear least squares implemented with Matlab’s fit function. Lower bounds were set on parameters to enforce any non-negative parameter values. Confidence intervals are calculated using the Matlab confint option. Using this method, confidence bounds are given by

$$ C = b \pm t \sqrt{S}, $$

where *b* is the parameter estimates, *t* is the quantile of Student’s *t* distribution with the model’s error degrees of freedom, and *S* is a vector of the diagonal elements from the estimated covariance matrix of the coefficient estimates, $(J^TJ)^{-1}s^2$, with *J* the Jacobian of the fitted values with respect to the parameters and $s^2$ the mean squared error. To aid with reproducibility, all Matlab code used to generate results is freely available on GitHub (https://github.com/brocksherlockmaths/Does-Timing-Matter).

#### Linear Model Effects

A linear model is one in which the dependent variable is a linear combination of the independent variables. That is any model of the form

$$\begin{aligned} Y = \sum _i \beta _iX_i, \end{aligned}$$

where $\beta _i$ are coefficients of the $X_i$ independent variables. For our purposes, we do not call a model linear if the coefficients are linear, e.g., we do not call $Y=\beta X^2$ linear, even though linear least squares can be applied to this problem for parameter inference.

Consider the linear model,

6 $$\begin{aligned} {Y_{j}} = \beta _0 + \beta _x{X_{j}} + \epsilon _{j}, \end{aligned}$$

where $\beta _0$ and $\beta _x$ are parameters and $\epsilon _j$ is the *error in the equation*, where subscript *j* denotes the *j*th observation. The error in the equation is when the model parameters do not capture all the output and the discrepancy is captured as epsilon. This represents the fact that we have a deterministic model and we do not believe the model to be a perfect representation of the data. In the statistics literature, a distinction is made between the error in the equation and the error in the measurement of the dependent variable *Y*. As the dataset consists of paired observations (*x*, *y*), the measurement of *Y* may have an inherent error separate to the error in the equation, $\epsilon$. Here we will assume that *Y* is measured without error, as is done through most of the measurement error literature. We highlight the distinction here as the mathematical biology inference literature tends to use $\epsilon$ to capture both possible dependent variable measurement errors and the error in the equation (Murphy et al. 2024). This is a natural consequence if the error in the measurement of *Y* is additive.

***Bias of the Slope*** Using linear least squares to estimate parameters provides a framework for some theoretical results. For non-controlled experiments with classical errors (errors of the form $W=X+U$), it has been shown that simple linear regression models underestimate the slope parameter (i.e., bias the slope towards zero) (Berkson 1950; Meites et al. 1984). Later studies showed the bias towards zero does not hold for multivariate linear regressions or nonlinear models (Hausman et al. 1995; Hausman 2001). Indeed, even for linear regression models, if the slope parameter is restricted to positive values, then the bias is away from zero (Meijer and Wansbeek 2000). A common method for correcting for measurement errors is the use of an instrumental variable (Schennach 2016). This requires the availability of a suitable auxiliary variable, the instrument, that is correlated with the independent variable but not its error. However, this method fails in the nonlinear case (Amemiya 1985).

This bias towards zero in the classical error case becomes clear when a least-squares fit of the linear model using the observed data *W* with the response *Y* is considered. Following (Carroll 2006; Buzas et al. 2004; Meites et al. 1984), this least-squares fit results in a consistent estimate not of $\beta _x$, but of $\beta _{x^*}= \lambda \beta _x$, where

7 $$\begin{aligned} \lambda = \frac{\sigma _x^2}{\sigma _x^2+\sigma _u^2}<1, \end{aligned}$$

with $\sigma _u^2$ is the variance of the measurement error and $\sigma _x^2$ is the variance of the independent variable. This is known as the reliability ratio. Note that since the variance of the measurement error $\sigma _u^2$ must be positive, the reliability ratio is less than one, biasing the slope estimate towards zero. If the reliability ratio can be estimated, then it can be used as a correction to the parameter estimate. We give more details of this in Section 4.

Another effect of measurement error in the independent variable is an increased variability of the data about the line of best fit. Substituting the classical error term, ${X} = {W} - {U},$ into Equation (6) yields the model

$$\begin{aligned} {Y}=\beta _0 + \beta _x {W} + (\epsilon - \beta _x {U}), \end{aligned}$$

with total error $(\epsilon - \beta _x {U})$ that has variance

$$\begin{aligned} \sigma _\epsilon ^2+\beta _x^2\sigma _u^2 > \sigma _\epsilon ^2. \end{aligned}$$

So, in the presence of measurement error there is an increase in variance of the *Y* data. Note that, the total error and the measurement of the independent variable, *W*, have a common component, *U*, which causes the bias in the estimate (Carroll 2006).

**Fig. 1:**
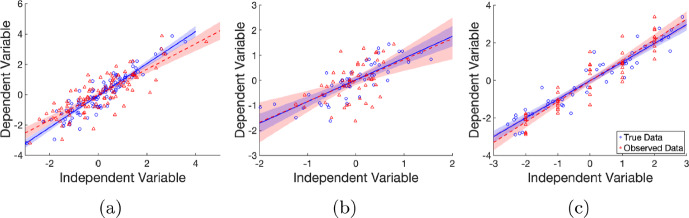
Fitting linear systems. Naive inference using least squares for **(a)** Non-controlled Experiment, Classical Error, **(b)** Non-controlled Experiment, Berkson Error, and **(c)** Controlled Experiment, Berkson Error, each for both true data (blue circles) and observed data (red triangles). The lines of best fit given the true data and the observed data are shown by the blue and dashed red lines respectively. Shaded regions in the corresponding colours indicate the 95% confidence interval bounds. Refer to Table 2 for the experiment protocols. All data is generated with true parameter values $\beta _x = 1$ and $\beta _0 = 0$. The estimated parameter values for each synthetic dataset are given in Table 3

In what follows, we design a sequence of experiments to illustrate the effect of measurement errors on the estimate of the slope parameter $\beta _x$ for the linear model given by Equation (6) with some numerical studies of synthetic data. Specifically, Figure 1 shows the effects on a linear least-squares fit on data with classical ($W=X+U$) and Berkson ($X=W+U$) type errors for controlled and non-controlled experiments. We fit the data with the observed independent variable values (*w*, *y*), and also with the true values (*x*, *y*). In each case we generate the *y* values using Equation (6) with the corresponding true independent variable value *x*. That is, *Y* data is always generated from the true underlying value, not what is observed. Each synthetic dataset contains 50 data points and the setup for each experiment and the distributions used are given in Table 2. Each dataset and their respective fits are shown in Figure 1. The parameter estimates and goodness of fit for each case are shown in Table 3. Note in particular that the fit for the non-controlled experiment data with Berkson errors was poorly constrained (low goodness of fit, adjusted $R^2$), although the estimated values were reasonable. In all setups, the quality of the fit to the observed data was poorer than the fit to the true data with all cases seeing an increase in the width of the confidence interval. Note that the case of a non-controlled experiment with Berkson error is the only case where the confidence interval for the slope parameter $\beta _x$ does not contain the true value. In the exemplars in Figure 1, it can be seen that the least squares estimate is biased for the classical error case. However, for both a controlled and non-controlled experiment, the estimate with Berkson error is closer to the true estimate – which is to be expected as estimates of the slope parameter for linear models under Berkson error are unbiased (Berkson 1950).

**Table 2:** Summary of protocols for the linear systems shown in Figure 1

Experimental design	Error type	Distribution of *W* (Berkson) or *X* (classical)	Distribution of *U*	Distribution of $\epsilon$
*Figure* 1*(a)*				
Non-controlled	Classical error $W = X + U$	$X \sim \mathcal {N}(0,\,1)$ observed	$U \sim \mathcal {N}(0,\,0.25)$	$\epsilon \sim \mathcal {N}(0,\,0.25)$
*Figure* 1*(b)*				
Non-controlled	Berkson error $X = W + U$	$W \sim \mathcal {N}(0,\,1)$	$U \sim \mathcal {N}(0,\,0.25)$	$\epsilon \sim \mathcal {N}(0,\,0.25)$
*Figure* 1*(c)*				
Controlled	Berkson error $X = W + U$	$W$ at 5 equally spaced points in $[2,2]$, 10 replicates each	$U \sim \mathcal {N}(0,\,0.25)$	$\epsilon \sim \mathcal {N}(0,\,0.25)$

**Table 3:** Parameter estimates and fit statistics – Linear System with true parameter values $\beta _x=1$ and $\beta _0=0$, showing the parameter estimates with 95% confidence intervals in brackets, and the goodness of fit, $R^2$ and the adjusted $R^2$, for the data sets with the different errors

	$\beta _x$		$\beta _0$		$R^2$	Adj $R^2$
*True Values*	1		0			
$Y = \beta _0 + \beta _x(W-U) + \epsilon$, *Non-Controlled Classical Error*						
True Data	1.058	(0.9707, 1.144)	-0.06248	(-0.1635, 0.03856)	0.8562	0.8548
Observed	0.8431	(0.7275, 0.9587)	-0.005443	(-0.1554, 0.1445)	0.6813	0.6781
$Y = \beta _0 + \beta _x(W+U) + \epsilon$, *Non-Controlled Berkson Error*						
True Data	0.8681	(0.6836, 1.053)	0.02067	(-0.1092, 0.1505)	0.6509	0.6436
Observed	0.8368	(0.4398, 1.234)	-0.005476	(-0.1927, 0.1817)	0.2723	0.2571
$Y = \beta _0 + \beta _x(W+U) + \epsilon$, *Controlled Berkson Error*						
True Data	0.9929	(0.9012, 1.085)	-0.01492	(-0.1614, 0.1315)	0.9081	0.9062
Observed	1.094	(0.9683, 1.22)	-0.02851	(-0.2065, 0.1495)	0.8643	0.8614

Note the poor fit (low $R^2$) for the fit to the non-controlled experiment with Berkson errors

***Variance of the Slope Estimate*** Another effect of measurement error is that the naive estimate of the slope using the observed data *W* can be less variable (tighter confidence intervals) than estimates derived from the true values *X*. In the case of classical errors, the variance of the estimator using *W* is less than the estimator using *X* asymptotically if and only if

8 $$\begin{aligned} \frac{\beta _x^2\sigma _x^2}{(\sigma _x^2+\sigma _u^2) }< \frac{\sigma _\epsilon ^2}{\sigma _x^2}. \end{aligned}$$

This inequality can be satisfied when the error in the equation variance, $\sigma _\epsilon ^2$, is small, the measurement error variance, $\sigma _u^2$, is large, or the magnitude of the gradient of the slope, $\vert \beta _x\vert$, is small (Buzas et al. 2004). Thus, estimating the slope from observed values *W* (with classical measurement error) can be more precise, yet biased, than when *X* is measured without error. This does not occur with Berkson errors, as the variance of the naive estimator is asymptotically never less than the variance of the true-data estimator.

**Fig. 2:**
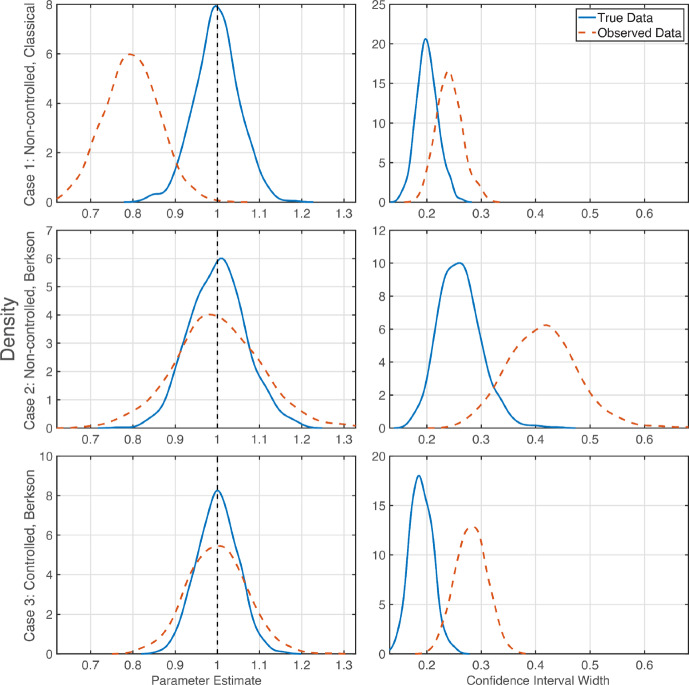
Slope estimation for linear systems with different errors. Densities of the slope parameter estimates (left) and confidence interval widths (right) from least squares fitting of naive model for classical and Berkson errors in non-controlled and controlled experiments for both true data (solid blue) and observed data (dashed red). Densities are produced from estimates of the slope from 1000 generated synthetic datasets. The synthetic data structures are as described in Table 2

To demonstrate the effects of measurement error under resampling of the data, we re-estimated the model parameters for each of the three scenarios described in Table 2 for 1000 generated datasets. The densities of the parameter estimates and confidence interval widths were generated with the built-in Matlab function ksdensity. The densities of the parameter estimate and the confidence interval width for the slope parameter from repeating the parameter estimation are shown in Figure 2. Due to our choice of error distributions we do not satisfy the inequality given in Equation (8) in this synthetic data study. Thus we do not observe the narrower confidence intervals for the error-contaminated observed data, *W*. Even so, other effects of measurement error are still observable.

We can see that in the systems with classical error the slope is consistently underestimated. This underestimation occurs without drastically increasing the width of the confidence interval – this can lead to an interpretation of being quite confident in the erroneous parameter estimate. With Berkson error, the slope estimate remains centred on the true value, albeit with slightly greater variance. The width of the confidence interval increases however, implying less confidence in the estimates. In the case of a controlled experiment with Berkson errors the confidence interval does not widen as much as for the non-controlled experiment with Berkson errors. From this, we learn that classical errors introduce bias to the slope estimate without significantly decreasing confidence, estimates are unbiased for Berkson errors (but with higher variance), and controlled experiments reduce the increase in the width of the confidence interval.

#### Nonlinear Model Effects

Although the effects of measurement errors on linear models are well understood, many systems are nonlinear. That is, the relationship between the dependent and independent variables cannot be expressed by Equation (6). Although there are no general analytical results that inform us of expected impacts of measurement error for nonlinear models we will present literature from some studies that observe their impacts.

A common approach to analysing nonlinear relationships is to apply a transformation to obtain an approximate linear form. For example, data following an exponential relationship,

9 $$\begin{aligned} y = A e^{-B x}, \end{aligned}$$

can be linearised by taking logarithms. In particular, the use of transforms is popular to remove heteroskedasticity and have the data follow standard assumptions (Box and Cox 1964). Indeed it has been found that, in some cases, transforming the data using logarithms can transform multiplicative error to additive error (Carroll 2006).

Transformations can also alter the properties of measurement errors in the dependent variable. Some articles suggest the transformation of nonlinear models to a linear model is not optimal for inference as experimental measurement errors can be distorted by the transformation (Motulsky and Ransnas 1987; Rhinehart 2016). For example, linearising the exponential decay in Equation (9) by taking logarithms enhances errors associated with observations with small dependent variable measurements *y*. As linear least squares is sensitive to outliers, these data points are given disproportionate weight in the fit – leading to a slope that is not an optimal estimate of the rate constant *B*. Note this only applies to functional transforms where the functional form and units are modified. The problem does not occur for linearising approximations (such as approximating a nonlinear term with a truncated Taylor series expansion) (Rhinehart 2016).

In this work, we investigate these effects in the context of synthetic numerical studies, where the independent variable is time. We generate synthetic data from given models and then introduce measurement errors – both in the dependent and independent variables – and assess the impact on parameter estimation. This allows us to evaluate the robustness of estimation procedures and compare the impacts across a range of models with a variety of measurement errors. In all our synthetic data studies, the dependent variable *y* is generated from the true independent variable *x* using a model $y = y(\theta , \epsilon , x)$, where $\theta$ is the model parameters, $\epsilon$ is the error in the equation and *x* is the true value of the independent variable. The resulting true data pairs are (*x*, *y*). However, in practice, the experimentalist observes only an error-prone version of the independent variable, *w*, and hence the available dataset is (*w*, *y*). Parameters are estimated from the observed data (*w*, *y*), and the estimates $\hat{\theta }$ are compared to the true values $\theta$ to study the effects of measurement error on inference. In some cases, we also compare the parameter estimates using (*w*, *y*) to those obtained from inference using the true data (*x*, *y*) to demonstrate that the bias in parameter estimates using (*w*, *y*) is the result of the measurement error and not due to the inference procedure itself – we see that better estimates are achieved using (*x*, *y*).

We investigate an oscillating system and three growth models below. In each investigation, we first demonstrate the synthetic data generation with errors and the fitting procedure for a single dataset. We then generate many realisations of the synthetic datasets, with random error, to analyse the effects of the error on inference across a range of data realisations. The synthetic data generated includes both biased and unbiased error models. We choose to analyse our synthetic data on the original scale (without any transformations) to avoid conflating effects of the measurement error with effects of the transformation.

Each investigation considers a different type of error, building from simplest to most complex.

First, the oscillating system considers:

- unbiased, normally distributed error terms for both dependent and independent variables.

The parasite model then introduces:

- multiplicative error for the dependent variable, and
- limits data to only a single observation per time point.

The tumour growth investigation considers:

- observations for multiple individuals at each time point, and
- a correlated error to the time measurements, where the error for each individual is dependent on the error from the other individuals.

Finally, the GLUT4 translocation investigation introduces:

- a biased error on the time variable, and
- Bayesian inference as a demonstration that measurement errors can influence our inference results for both a frequentist and Bayesian approach.

In each investigation, both exemplars of the parameter estimates for a single data realisation and the densities of parameter estimates for multiple data realisations are explored.

In Section 4 we will see methods, in both frequentist and Bayesian settings, that can be introduced to account for measurement errors.

***An Oscillating System*** We visualise the impact of measurement errors on nonlinear models by first considering the case of a simple oscillating model:

10 $$\begin{aligned} Y = a\cos {(bX)} + \epsilon , \end{aligned}$$

where *a* is the amplitude and *b* controls the frequency of oscillation. This simple model is chosen for interpretability of its parameters, noting that oscillating systems are common in the biological sciences; see for example (Hancock et al. 2017; Wu 1996; Robinson et al. 1952; Oladepo and Peace 2025).

We consider various combinations of experimental design and error types, namely classical errors for non-controlled experiments and Berkson errors for controlled and non-controlled experiments. We highlight that a main point of difference between the non-controlled and controlled experiments is that the non-controlled experiments have only one observation per independent variable value; the controlled experiments have multiple observations per independent variable value. This is a consequence of sampling the independent variable values from a distribution as opposed to setting the values by design. We refer to the fixed times in the controlled experiment as *protocol times*.

The distributions for each experiment are summarised in Table 4 where the generation of true values *X* and observed values *W* is given. Here, the errors in the dependent and independent variables are of the same magnitude. Synthetic datasets are generated using Equation (10) with true parameter values taken to be $a=3$ and $b=4$. Recall the dependent variable data *y* is always generated from the underlying true independent variable value *x*. We then have two datasets for each experimental setup: the true data pairings (*x*, *y*) and the observed data pairings (*w*, *y*).

Each dataset consists of 50 data points. For the non-controlled experiment, this specifically implies that we take 50 randomly chosen independent variable values from the distributions given in Table 4. We consider two cases for the controlled experiment, one where observations are taken at linearly spaced points not aligned with the turning points in the oscillation and a second case where all observations are aligned to turning points (so the amplitude is directly observed). In both controlled cases, there are five designated protocol times, each with ten associated observations. That is, there are five unique values *w*, each replicated ten times and each replicate receives its own random error such that there are 50 unique true values *x*.

Each dataset (both (*x*, *y*) and (*w*, *y*) pairings) is fit with the model $y(x)=a\cos (bx)$ using nonlinear least squares. In the fitting we assume the frequency, *b*, is known and we only attempt to infer the amplitude, *a*.

**Table 4:** Summary of the protocols for the oscillatory systems shown in Figure 3

Experimental design	Error type	Distribution of *W* (Berkson) or *X* (classical)	Distribution of *U*	Distribution of $\epsilon$
*Figure* 3*(a)*				
Non-controlled	Classical error $W = X + U$	$X \sim \mathcal {U}(-2,\,2)$	$U \sim \mathcal {N}(0,\,0.05)$	$\epsilon \sim \mathcal {N}(0,\,0.05)$
*Figure* 3*(b)*				
Non-controlled	Berkson error $X = W + U$	$W \sim \mathcal {U}(-2,\,2)$	$U \sim \mathcal {N}(0,\,0.05)$	$\epsilon \sim \mathcal {N}(0,\,0.05)$
*Figure* 3*(c)*				
Controlled	Berkson error $X = W + U$	*W* at 5 equally spaced points on $[-2,\,2]$	$U \sim \mathcal {N}(0,\,0.05)$	$\epsilon \sim \mathcal {N}(0,\,0.05)$
*Figure* 3*(d)*				
Controlled	Berkson error $X = W + U$	*W* at 5 equally spaced points on $[-\pi /2,\,\pi /2]$	$U \sim \mathcal {N}(0,\,0.05)$	$\epsilon \sim \mathcal {N}(0,\,0.05)$

**Fig. 3:**
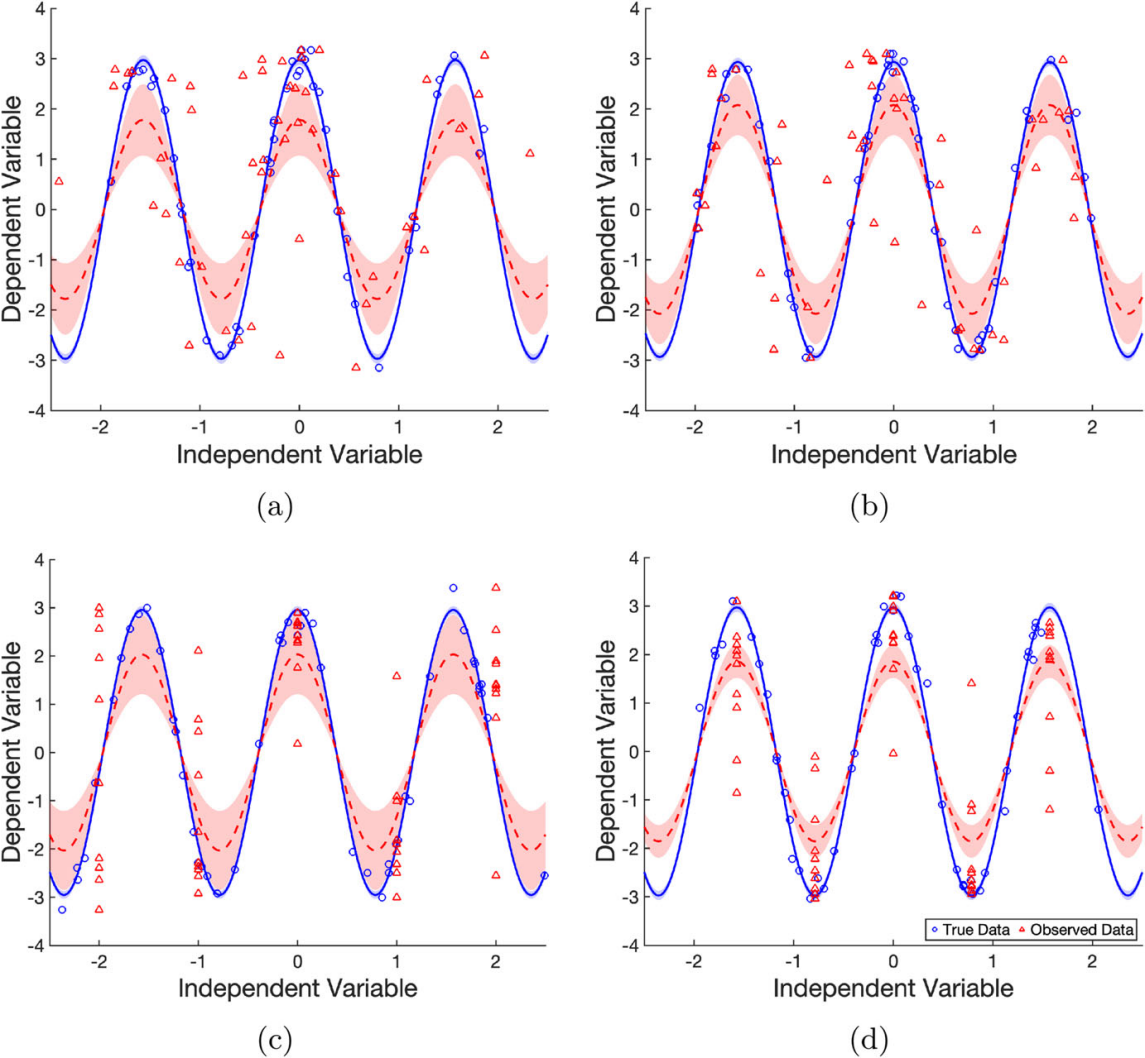
Fitting oscillatory systems. Model outputs using naive estimates of the amplitude parameter for **(a)** non-controlled experiment, classical error, **(b)** non-controlled experiment, Berkson error, **(c)** controlled experiment, Berkson error (observation points are linearly spaced on $[-2,2]$), and **(d)** controlled experiment Berkson error (observations linearly spaced on $[-\pi /2,\pi /2]$), each for both true data *X* (blue circles) and observed data *W* (red triangles). The model outputs with optimised parameter values are shown by solid blue and dashed red lines for the true data and observed data respectively. Shaded regions in the corresponding colours indicate the 95% confidence interval bounds

The effects of measurement error on the parameter estimates for an oscillating system are shown in Figure 3. The parameter estimates and goodness of fit are shown in Table 5. In all the cases investigated the amplitude is underestimated in the presence of measurement error, regardless of error type or experimental control. When fitting to the observed data (*w*, *y*), the confidence interval does not contain the true value of the amplitude regardless of the setup. This is in contrast to the fits of the true data (*x*, *y*) which contain the true value in the confidence interval for each case. The goodness fit is consistently poorer when the observed data is used, in some cases significantly so. Note that the goodness of fit is least impacted by the measurement error in the controlled Berkson case with measurements aligned to the turning points of the oscillation – though it is still poorer than the true data case. In the worst case, for the observed data of the non-controlled experiment with classical error, the adjusted $R^2$ value is 0.2020 indicating a poor fit. The poor fit is also reflected by the inferred value for *a* in this case – the 95% confidence intervals do not contain the true value.

Recall that a masking of features for nonlinear models was observed in Carroll (2006). For an oscillating model, this refers to the oscillations being masked by the measurement error in such a way that, given the data alone, an oscillating model may not be chosen for the system. We can observe slight masking in Figures 3a and 3b. This masking occurs for each protocol if sufficient measurement error is introduced. Notably even in Figure 3d, the controlled experiment where observations coincide with turning points (and the positions carrying the most direct information about amplitude), the fitted amplitude is also underestimated. This systematic underestimation arises because the measurement error only compresses the observed range; it cannot produce values beyond the true amplitude.

**Table 5:** Parameter estimates and fit statistics – Oscillating System with true parameter values $a=3$ and $b=4$, (where *b* is fixed and not inferred), showing the parameter estimates with 95% confidence intervals in brackets, and the goodness of fit, $R^2$ and the adjusted $R^2$, for the data sets with the different errors

	*a*		$R^2$	Adj $R^2$
*True Value*	3			
*Equation* (10), *Non-Controlled Classical*				
True Data	2.972	(2.878, 3.067)	0.9852	0.9852
Observed	1.782	(1.072, 2.492)	0.2020	0.2020
*Equation* (10), *Non-Controlled Berkson*				
True Data	2.934	(2.852, 3.017)	0.9897	0.9897
Observed	2.077	(1.476, 2.679)	0.4535	0.4535
*Equation* (10), *Controlled Berkson (Case 1)*				
True Data	2.954	(2.874, 3.033)	0.9913	0.9913
Observed	2.032	(1.21, 2.854)	0.3313	0.3313
*Equation* (10), *Controlled Berkson (Case 2)*				
True Data	2.973	(2.88, 3.066)	0.9881	0.9881
Observed	1.856	(1.516, 2.195)	0.7068	0.7068

**Fig. 4:**
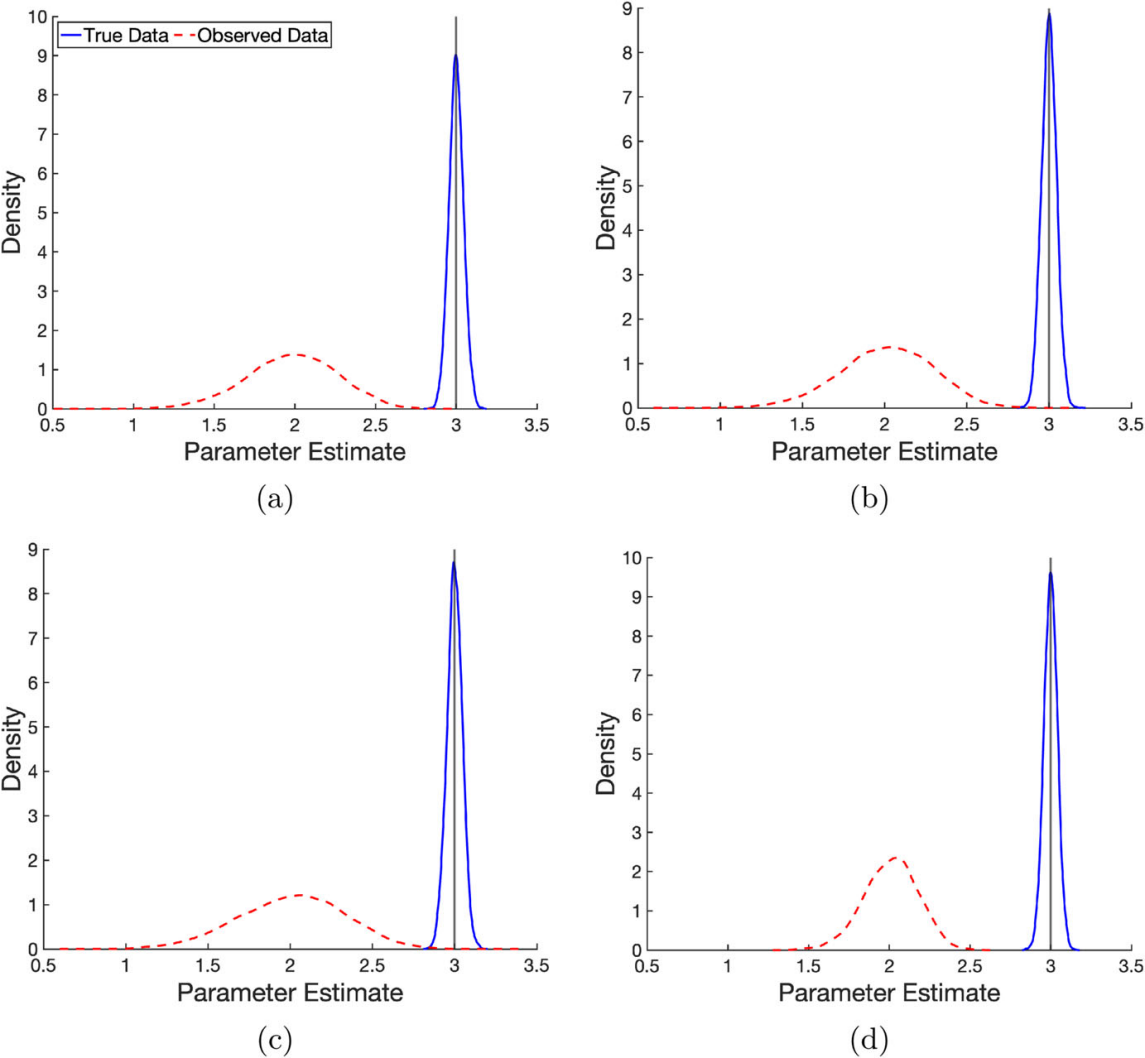
Amplitude estimation for oscillatory systems. Densities of amplitude parameter estimates from least squares fitting of naive model for classical and Berkson errors in non-controlled and controlled experiments for both true data (solid blue) and observed data (dashed red). Densities are produced from estimates of the amplitude from 10000 generated synthetic datasets. In controlled experiment **(a)**, observation points are linearly spaced on $[-2,2]$ with a gap of one between each observation point. The controlled experiment **(b)** has a gap of $\pi /2$ between each observation point and is linearly spaced on $[-\pi /2,\pi /2]$

We now examine if these observations are consistent when the parameter estimation is repeated across resampled datasets. Each experiment above was repeated for 10000 synthetic datasets, generated using the same parameter values and error distributions described previously in Table 4. The resulting amplitude-estimate densities – computed using the built-in MATLAB function ksdensity – are shown in Figure 4. These results show that measurement error in the independent variable leads to a consistent underestimation of the amplitude, irrespective of error type or experiment under resampling.

***Parasite Growth*** As a second non-linear example, we examine a parasite-growth model to demonstrate how misreporting the time of measurement affects parameter inference, when there is only a single observation per time point. We implement the model from Rebelo et al. (2021):

11 $$\begin{aligned} P(t)=V_0e^{gt} + B_0e^{-kt}, \end{aligned}$$

where *P*(*t*) is the number of parasites in culture at time *t*, which grow exponentially at rate *g*. The initial background fluorescence, $B_0$ (which prevents the initial parasite numbers from being observed directly) decreases at rate *k*. For this type of data, generally the aim is to infer an initial parasite level $V_0$. The original study (Rebelo et al. 2021) considered parasite samples taken from multiple subjects at multiple time points, which were then cultured and measured. Here, we simplify the setting to a single sample from a single patient to isolate the effects of measurement error in the simplest case.

Here synthetic data is generated using Equation (11) with actual measurement times *t* giving the dependent measurements *y*. Time measurements are subject to Berkson error: the true measurement time *t* is the protocol time *W* with an additive error $U \sim \mathcal {N}(0,\sigma _U^2)$ where $\sigma _U = 0.25$. In other words, the actual set of measurement times are ($t = W + U$).  Protocol observation days are $W=\{0,2,\dots ,24\}$ (every second day until day 24). This models the scenario where the measurement is taken at a random time during the day, but only the protocol day is recorded.

In this synthetic study, we assume there is no error in the equation ($\epsilon =0)$; in other words the number of parasites is able to be measured precisely and the only error is in the independent variable (the timing of the measurement). The true parameter values are taken to be: $V_0=0.002$, $g=0.531$, $B_0=0.02$, and $k=0.323$ (from (Li 2024)).

**Fig. 5:**
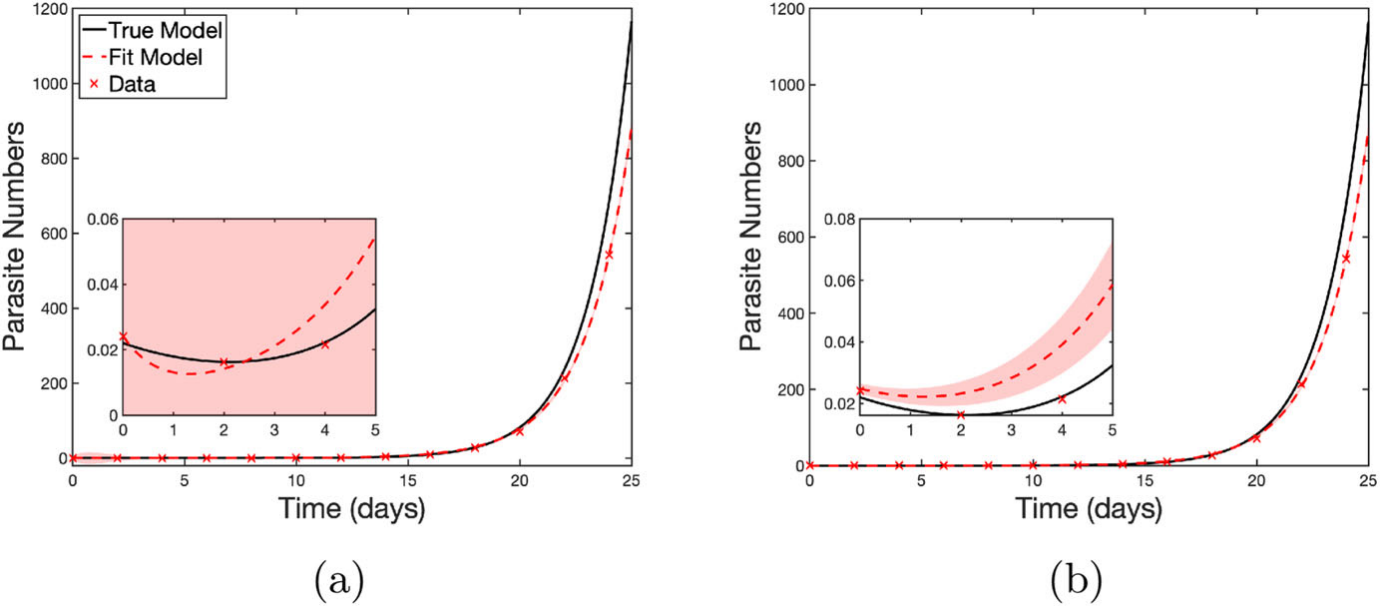
Fitting the parasite growth model, Equation  11. The synthetic data generated with measurement error is fitted using least-squares. Shaded regions indicate the 95% confidence interval bounds. The confidence intervals can be seen on both the large image and in better detail in the inset. Note that for the inset in **(a)** the confidence bounds are the entire region of the inset. **(a)** Results for a fit of all model parameters. **(b)** Results of a fit for $V_0$ and *g* with all other parameters set to their true values. Inset figures show the detail of the growth for the first five days

We consider numerical experiments where we fit the observed data (*w*, *y*) with least squares; the true measurement time *t* is treated as unknown. In the first scenario, all four parameters ($V_0,\,g,\,B_0,\,k$) are estimated simultaneously, Figure 5a. The parameter estimates for this instance were $V_0=0.004317$, $B_0=0.01656$, $g=0.4903$, and $k=1.224$. Note that the initial parasite $V_0$ estimate is more than twice the true value. In the second scenario, using the same data points, the background-fluorescence parameters $(B_0,\,k)$ are assumed to be known, and only $V_0$ and $g$ are inferred, Figure 5b. In this instance the initial parasite level was overestimated at $V_0=0.004845$, or 2.4 times the true $V_0$.

The full set of parameter estimates and goodness of fit for each case are shown in Table 6. Unsurprisingly the background fluorescence (parameters $B_0$ and *k*) is ill-constrained, with the confidence interval for this estimate containing zero even for the case with no error on the time variable – recall the error in the equation $\epsilon$ is zero, so even with perfect data these positive parameters are not identifiable. Note that the background fluorescence decays to zero, as seen in Equation 11, so there are few data points constraining these parameters. Although the goodness of fit ($R^2 \approx 1$) in all cases, the width of the confidence interval increases significantly in the case of measurement error and all parameters being fit. When only fitting $V_0$ and *g* with measurement error, the confidence intervals do not contain the true values.

**Table 6:** Parameter estimates and fit statistics – Parasite Growth, Equation (11), with true parameter values $V_0=0.002$, $g=0.531$, $B_0=0.02$, and $k=0.323$, showing the parameter estimates with 95% confidence intervals in brackets, and the goodness of fit, $R^2$ and the adjusted $R^2$ (4 significant figures), for the data with no time error, when all parameters were fitted to data with timing errors, and when only $V_0$ and *g* were fitted

$V_0$		*g*		$B_0$		*k*		$R^2$	Adj $R^2$
*True values*									
0.002		0.531		0.02		0.323			
*Equation* (11), *No Time Error*									
[1.986	(1.917,2.056)]E-3	0.5313	(0.5298,0.5328)	0.1752	(-0.4506,0.801)	0.1172	(-0.6401,0.8745)	1.0000	1.0000
*Equation* (11), *fitting all parameters with timing errors*									
[4.846	(3.01,6.681)]E-3	0.4845	(0.4685,0.5005)	0.0193	(-8.314,8.353)	1.314	(-2963,2965)	0.9996	0.9994
*Equation* (11), *fitting only* $V_0$ *and* *g* *with timing errors*									
[4.845	(3.230,6.461)]E-3	0.4845	(0.4704,0.4986)	0.02	fixed	0.323	fixed	0.9996	0.9995

Note that the fit for all the parameters with timing errors was poor – although the $R^2$ is high, the confidence intervals are very wide and contain zero in two cases, showing that these (positive) parameters are not identifiable

Having illustrated the effect of measurement error for a single realisation of the error, Figure 5, we next quantify the behaviour for a variety of error variances. We introduce an unbiased multiplicative error in the equation: $\epsilon \sim \mathcal {N}(0,\sigma _\epsilon ^2)$, so that our data is generated with

12 $$\begin{aligned} y = (1 + \epsilon )P(t). \end{aligned}$$

To systematically quantify the effects of the variance of the measurement error in both the independent and dependent variables ($\sigma _U^2$ and $\sigma _\epsilon ^2$, respectively) on estimates of the growth rate *g* and initial value $V_0$, we construct a grid comprising 20 linearly spaced points for each of $\sigma _\epsilon \in [0,\,0.5]$ and $\sigma _U \in [0,\,0.5]$. For each $(\sigma _U, \sigma _\epsilon )$ pair, we generate 1000 synthetic datasets using model (12) with the true parameter values $(V_0,\,g,\,B_0,\,k)$ above (consistent with Li (2024)). The upper bound of this grid corresponds to deliberately extreme and unrealistic error magnitudes. For instance $\sigma _U = 0.5$ corresponds to a standard deviation in timing of half a day, making it plausible that a true measurement time could occur on a different calendar day than the protocol time. We acknowledge that this is not realistic, but take these extreme values to better visualise the effects of the measurement error and highlight the robustness of inference to reasonable errors.

**Fig. 6:**
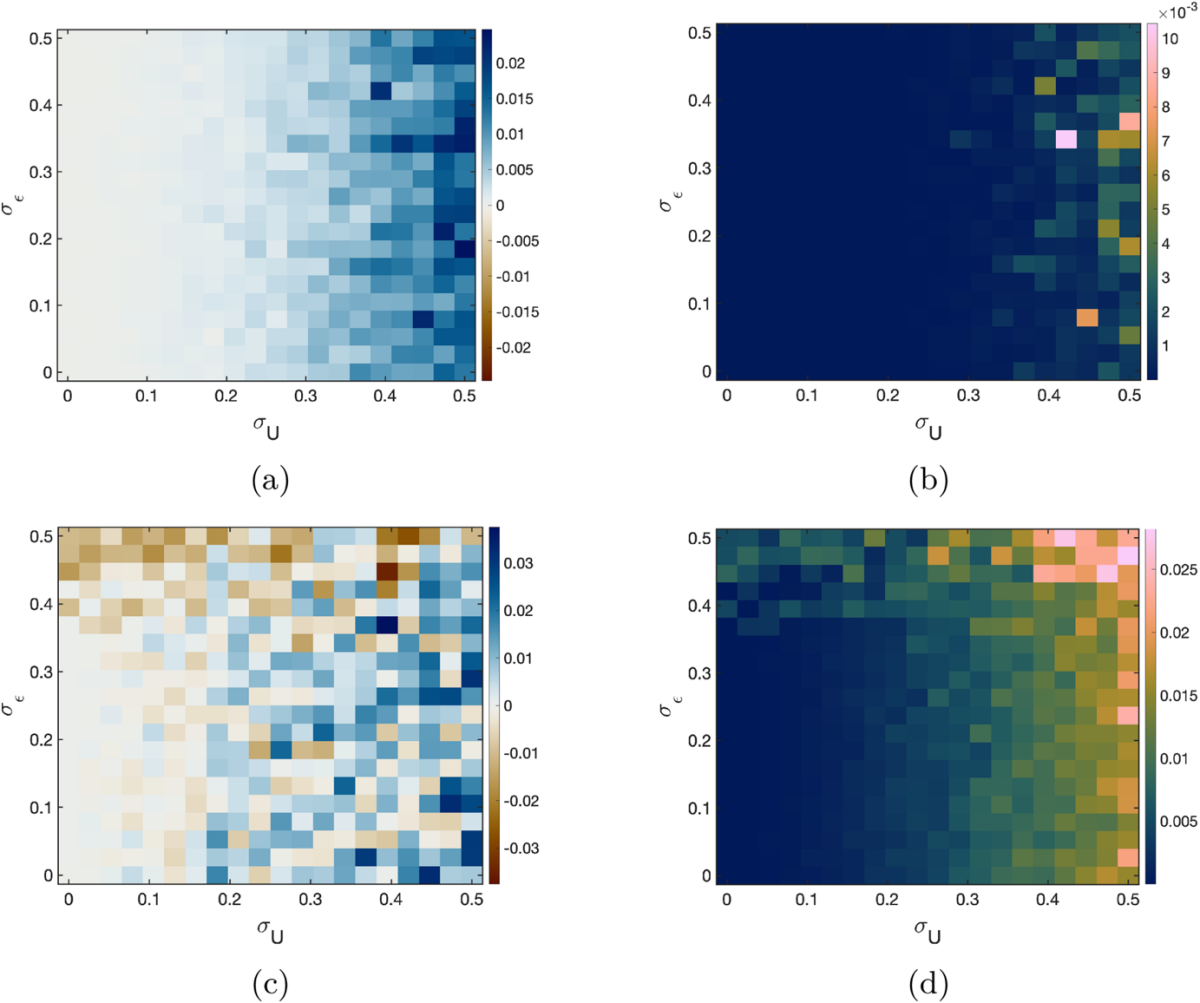
Bias and variance of $V_0$ and *g* estimates as functions of the standard deviation of the dependent measurement noise $\sigma _\epsilon$ and the standard deviation of the time measurement error $\sigma _U$. Synthetic data is generated using model (12). Time measurement errors are subject to Berkson error such that the true measurement time *t* is given by $t=W+U$, where *W* is the protocol time and the error is $U \sim \mathcal {N}(0,\sigma _U^2)$. Parameter estimates are found by fitting model (11) to 1000 realisations of synthetic data for each combination of error $\sigma _\epsilon$ and $\sigma _U$. For each combination of errors, the mean bias in the parameter estimates and the variance in the parameter estimates is calculated. Heatmaps show **(a)** Bias in $V_0$ estimate, **(b)** Variance of $V_0$ estimates, **(c)** Bias in *g* estimate, and **(d)** Variance of *g* estimates

We infer the initial parasite level and parasite growth rate for each dataset of (*w*, *y*) observations, giving 1000 paired estimates of $\hat{V_0}$ and $\hat{g}$ for each $(\sigma _U,\sigma _\epsilon )$ combination. From these we calculate the mean difference between our estimated parameter values ($\hat{V_0}$ and $\hat{g}$) and the true values ($V_0$ and *g*) and the variance of the estimated parameters for each $(\sigma _U,\sigma _\epsilon )$ combination. Figure 6 presents these results as four heatmaps, with $\sigma _U$ on the horizontal axis, $\sigma _\epsilon$ on the vertical axis, and the colour indicating the mean difference between the inferred and true values (Figure 6a and c) and the variance of the estimates (Figure 6b and d).

The heatmaps show that the estimates of $V_0$ are relatively robust to the error in the equation, but even moderate timing errors ($\sigma _U \gtrsim 0.2$) can bias the parameter estimates. Somewhat counterintuitively, although the measurement error is unbiased there is a bias in the parameter estimates such that the parameters tend to be overestimated when measurement error is present. The estimate of $V_0$ is relatively insensitive to increases in the variance of the equation error $\sigma _\epsilon ^2$, but does increase for large variances of the measurement error $\sigma _U^2$. In contrast, estimates of the growth rate *g* become biased for smaller variances of the measurement errors and are more sensitive to error in the variance of the equation error. The variance of the estimates of *g* increases when either $\sigma _\epsilon$ or $\sigma _U$ increases, suggesting that noise in both the independent and dependent variables has a compounding effect on growth-rate uncertainty. We see here that measurement error is the primary driver in the bias of parameter estimates when only a single observation is made at each protocol time.

***Tumour Growth*** The previous parasite example considered only a single observation per protocol time. We now turn our attention to an example with multiple observations per protocol time. In biological data, multiple measurements at a single time-point are often from different specimens, and the data collected from these specimens is often heterogeneous. For example, an experiment observing tumour growth across time will observe the growth of different tumours across a population of subjects – these different tumours then make up the repeat observations per time point. Each of these tumours likely has different intrinsic parameter values, for example their growth rates. The heterogeneity observed is not due to some error about the equation but due to the different behaviour of the subjects – for example, differing genetics between subjects. A common modelling approach is to treat all subjects as varying around some average behaviour. For this synthetic data study, we investigate the impacts of measurement error when tumour size is measured exactly, and heterogeneity in the data comes from parameter values differing between subjects.

For this case study, our synthetic data follows the experimental setup in Kim et al. (2011). Data of this form has been widely studied in the literature with a range of tumour growth models; see for example (Sherlock and Coster 2023; Jenner et al. 2018, 2019). We model the tumour growth using the Gompertz equation (Gompertz 1825; Kuang et al. 2016; Laird 1964),

$$\begin{aligned} \frac{dy(t)}{dt} = ry(t)\ln \left( \frac{K}{y(t)}\right) , \end{aligned}$$

where *y*(*t*) is the tumour volume ($\text {mm}^3$) at time *t*, *r* is the growth rate and *K* is the carrying capacity (i.e., the maximum size the tumour can grow, $K=\lim _{t \rightarrow \infty }y(t)$). The Gompertz growth model has an analytic solution given by

13 $$\begin{aligned} y(t) = K \left( \frac{y(0)}{K}\right) ^{\exp {(-rt)}}. \end{aligned}$$

Synthetic data was generated from Equation (13) with parameter values $y(0)\sim \mathcal {U}(100,120)$, growth rate $r\sim \mathcal {N}(0.08,0.02^2)$, and $K=4000$ for each tumour. Each synthetic dataset consists of six tumours – each with their own realisation of the parameters – with measurements taken every second day for 60 days. That is, the set of protocol times is $W = \{0,2,4,\dots ,60\}$. Least-squares fitting was undertaken to optimise the model parameters. All parameter fitting is done simultaneously to all tumours.

**Fig. 7:**
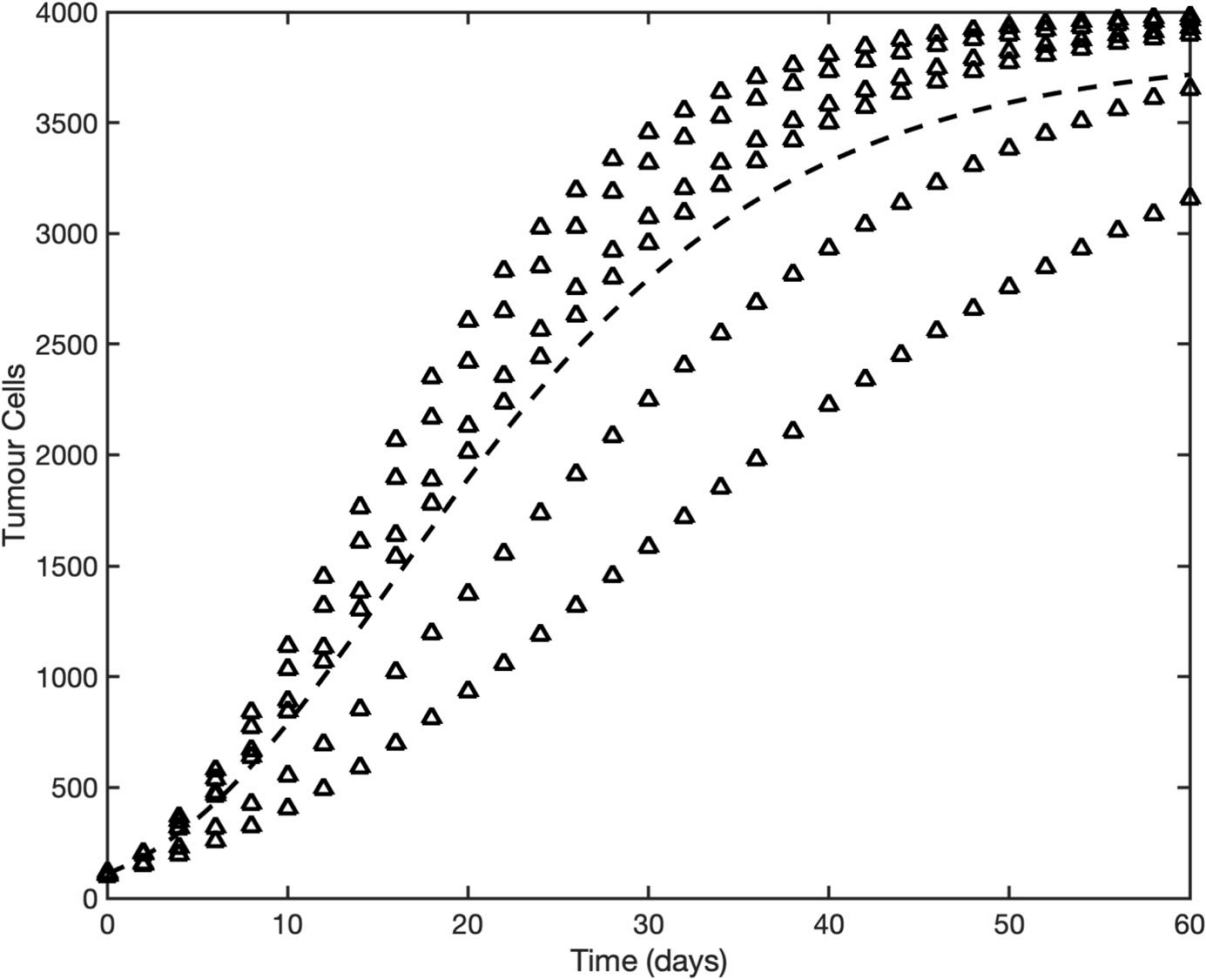
Model 13 is fit to synthetic data for tumour growth without measurement error. Synthetic data is generated with parameter values $y(0)\sim \mathcal {U}(100,120)$, growth rate $r\sim \mathcal {N}(0.08,0.02^2)$, and carrying capacity $K=4000$. Data consists of six tumours with measurements taken every second day for 60 days (data for each of the six tumours is shown by triangles). The growth rate, *r*, and carrying capacity, *K*, are inferred whilst the initial condition, *y*(0) was set to be the mean of the initial data points. Fit parameter values are $r=0.08082$ and $K=3821$. The model output with optimised parameters is shown by the dashed line

Figure 7 shows the data for six tumours without measurement error (measurements are taken at exactly the same time on each protocol day, so $t=W$) alongside the model output from the optimised parameter values. Only the growth rate *r* and carrying capacity *K* are estimated with the initial tumour size *y*(0) set to the mean of the data points at time $t=0$.

The optimised growth rate has a relative error (given by $(\hat{r} - r)/r$) of 0.0103 compared to the true mean value 0.08, indicating that the mean growth rate can be recovered with reasonable accuracy even with only six tumours. The estimated carrying capacity (3821) is in this case lower than the true value (4000). This discrepancy is to be expected because the tumours are not observed for a sufficiently long time to observe the trend of them reaching their carrying capacity.

We now introduce a correlated measurement error to the data. We have modelled measurement errors in a way that captures the scenario of a lone experimentalist who must measure the size of each tumour on the designated measurement days. Because only one tumour can be measured at a time and each measurement requires some varying duration, the observations are taken sequentially over the course of the day. However, as mentioned previously, typically only the protocol time is recorded, not the actual measurement times. Two forms of correlated measurement error are considered: *ordered error*, in which tumours are always measured in the same sequence, and *random-order error*, in which the measurement sequence varies across days.

Let $U_{ij}$ be the measurement error for the *i*th tumour at the *j*th protocol time. For sequential measurements the error arises from the time needed for the *i*th tumour to be measured on the *j*th day, $M_{ij}$, which is taken to be iid and lognormally distributed, i.e.,

14 $$\begin{aligned} M \sim Lognormal(\mu ,\sigma ^2), \end{aligned}$$

where $(\mu ,\sigma ^2)$ are the log–scale parameters. Setting the mean measurement duration $\mu _M$ with variance $\sigma _M^2$ for $M_{ij}$ on the original scale, we have

$$\begin{aligned} \mu = \ln \!\left( \frac{\mu _M^2}{\sqrt{\mu _M^2 + \sigma _M^2}}\right) , \qquad \sigma ^2 = \ln \!\left( 1 + \frac{\sigma _M^2}{\mu _M^2}\right) . \end{aligned}$$

For this study, we set $\mu _M=1/6$ and $\sigma _M=0.1$. The mean of $\mu _M = 1/6$ corresponds to an average of four hours per measurement, an extreme value deliberately chosen to demonstrate the lack of effect of the measurement error in this setting.

In the ordered error scenario, the experimentalist measures tumours in a fixed sequence each day: tumour 1 first, then tumour 2, and so on until all tumours are measured. So, the measurement error for tumour *T* on the *j*th day is given by

15 $$\begin{aligned} U_{Tj} = \sum _{i=1}^T m_{ij}, \end{aligned}$$

for all *j*. As a result, the true time of measurement $t_{ij}$ for the *i*th tumour on the *j*th day is $t_{ij} = W_j + U_{ij}$.

In the random-order error scenario, the experimentalist selects a different measurement sequence on each observation day. For example, on day 0, tumour 5 may be measured first, whereas on day 6, tumour 2 is measured first. As in the ordered-error case, we calculate the timing error for each tumour from the cumulative measurement times of the tumours measured earlier that day, but the ordering is randomly shuffled for each day.

**Fig. 8:**
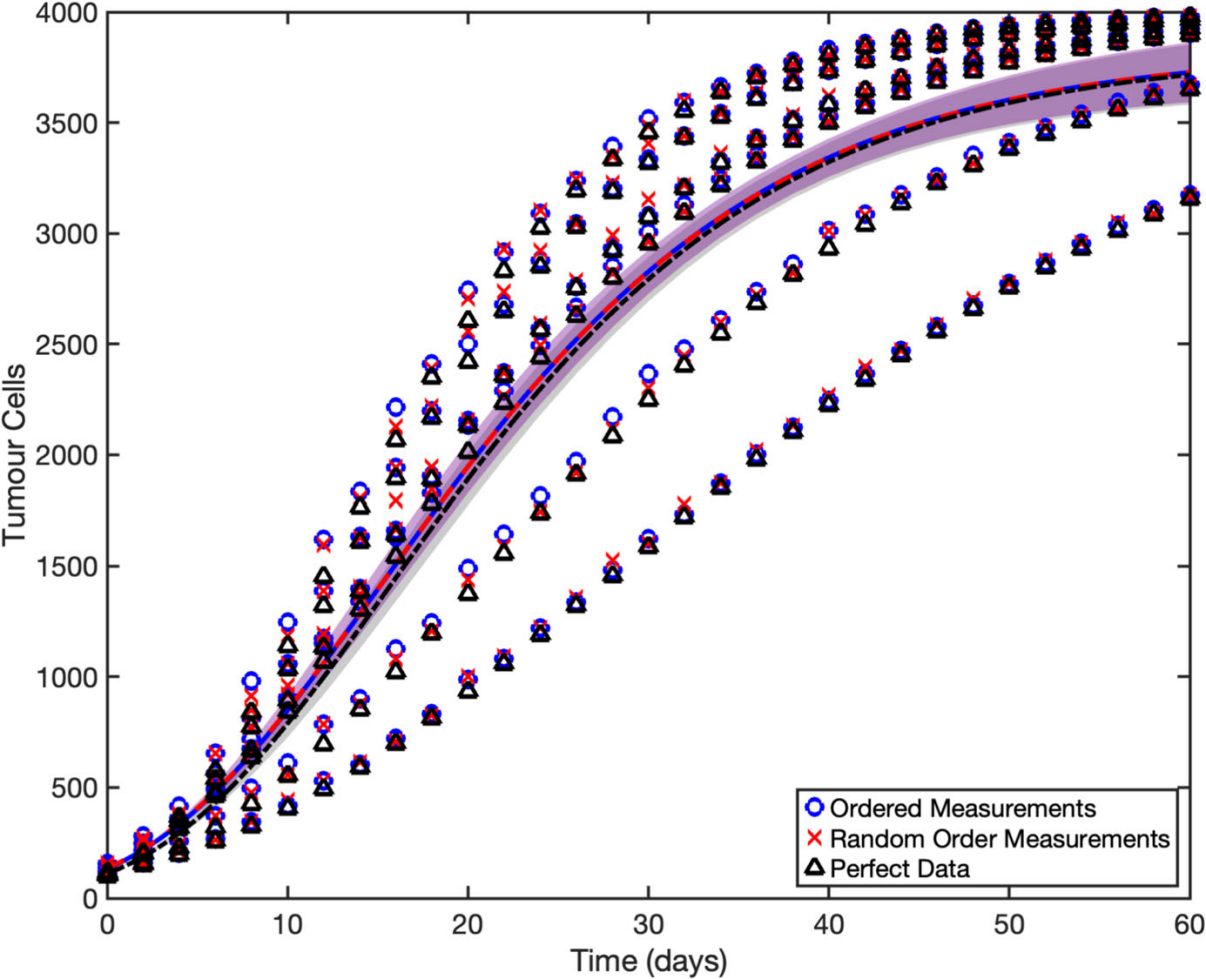
Fitting tumour growth. Model 13 is fit to synthetic data generated with ordered measurements, random order measurements, and without measurement error with $y(0)\sim \mathcal {U}(100,120)$, growth rate $r\sim \mathcal {N}(0.08,0.02^2)$, and carrying capacity $K=4000$. Data consists of six tumours with measurements taken every second day for 60 days. The growth rate, *r*, and carrying capacity, *K*, were inferred whilst the initial condition, *y*(0) was set to be the mean of the initial data points. Optimised parameter values were: $y(0)=107.39$, $r=0.08134$ and $K=3816$; $y(0)=133.13$, $r=0.08012$ and $K=3830$; and $y(0)=135.22$, $r=0.07987$ and $K=3833$, for the perfect data, ordered measurements, and random order measurements respectively. Shaded regions in the corresponding colours indicate the 95% confidence interval bounds for the different cases. Note that the interval bounds almost completely overlap

Figure 8 shows the model outputs obtained by fitting the Gompertz model, Equation (13), to synthetic data ((*w*, *y*) observations as the true values (*t*, *y*) are unknown) for each measurement error scenario. In all cases, the model outputs are in close correspondence, even with data with large measurement errors. The largest discrepancies occur at early time points, with outputs converging as time advances. The parameter values from the fits and the fit statistics are given in Table 7. The goodness of fit remains relatively consistent across all scenarios. Indeed, there is no significant change in the confidence intervals across the scenarios and the fits appear robust to the measurement error. The only appreciable change is an increase in the carrying capacity estimates and of the initial condition *y*(0) compared to the perfect data case, though this is expected as all measurement errors are delayed measurements. The system is strictly increasing, so these delay measurements result in higher initial values. Similar results are observed when the initial condition *y*(0) is included in the fit routine. We see that the initial condition is consistently overestimated across all three scenarios. However, the overestimation is greater when measurement error is present.

**Table 7:** Parameter estimates and fit statistics – Tumour Growth with true mean parameter values $r=0.08$, $K=4000$, and $y(0)=110$, showing the parameter estimates with 95% confidence intervals in brackets, and the goodness of fit, $R^2$ and the adjusted $R^2$, for the perfect, ordered measurement, and random order measurement data sets

	*r*		*K*		*y*(0)		$R^2$	Adj $R^2$
*True Values*	0.08		4000		110			
*Equation* 13, *y*(0) *fixed to mean*								
Perfect	0.08082	(0.07464, 0.087)	3821	(3651, 3991)	110.9	fixed	0.8706	0.8699
Ordered	0.08012	(0.07385, 0.08639)	3830	(3661, 4000)	133.1	fixed	0.8688	0.8681
Random	0.07987	(0.07358, 0.08615)	3833	(3662, 4003)	135.2	fixed	0.8680	0.8673
*Equation* 13, *All Parameters*								
Perfect	0.07838	(0.06483, 0.09193)	3846	(3630, 4061)	128.2	(37.23, 219.2)	0.8707	0.8693
Ordered	0.07823	(0.06478, 0.09168)	3849	(3637, 4061)	147.9	(50.29, 245.6)	0.8688	0.8674
Random	0.07823	(0.06474, 0.09172)	3849	(3637, 4061)	148.1	(50.13, 246.2)	0.8681	0.8666

**Fig. 9:**
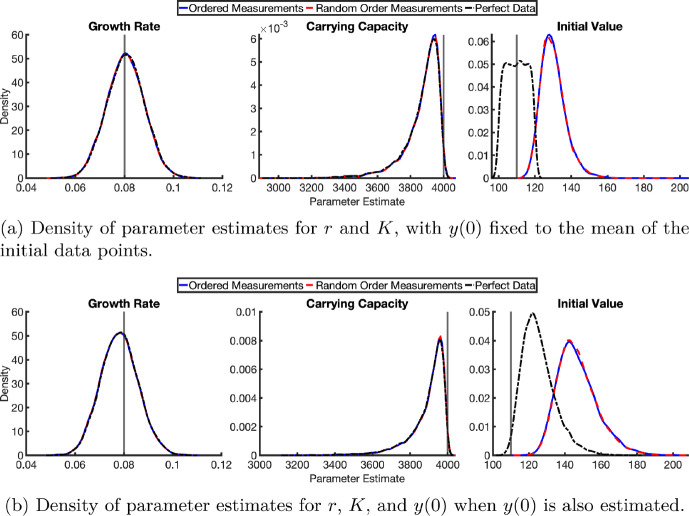
Parameter estimation for tumour growth from 10000 synthetic datasets. Synthetic data were generated with $y(0)\sim \mathcal {U}(100,120)$, $r\sim \mathcal {N}(0.08,0.02^2)$, and $K=4000$ for six tumours, with measurements taken every second day for 60 days. Each panel shows the density of parameter estimates from least squares fitting with **(a)** setting *y*(0) fixed to the mean of the data at time zero, and **(b)** including *y*(0) in the fit routine

The fitting routine was repeated for 10000 synthetic datasets (repeating the measurements of the six tumours) to ensure results were not the outcome of a particular realisation of the parameters and error. The densities of the resulting estimated parameters are shown in Figure 9a and b, for the cases where the initial value was fixed to the mean of the data at time zero and where it was additionally estimated within the fitting routine, respectively. In both cases, measurement error has no detectable effect on the estimated growth rate, which remains unbiased. The carrying capacity is in this case consistently underestimated – again, as is expected given that the observation window ends before the tumour reaches its carrying capacity. More importantly, the density profiles for the true and measurement-error data are nearly indistinguishable, except for the initial tumour size. The initial size is consistently overestimated when there is measurement error. When the initial condition is included in the fit routine (Figure 9b) then overestimation also occurs for the true data, albeit to a lesser extent than with measurement error.

**Fig. 10:**
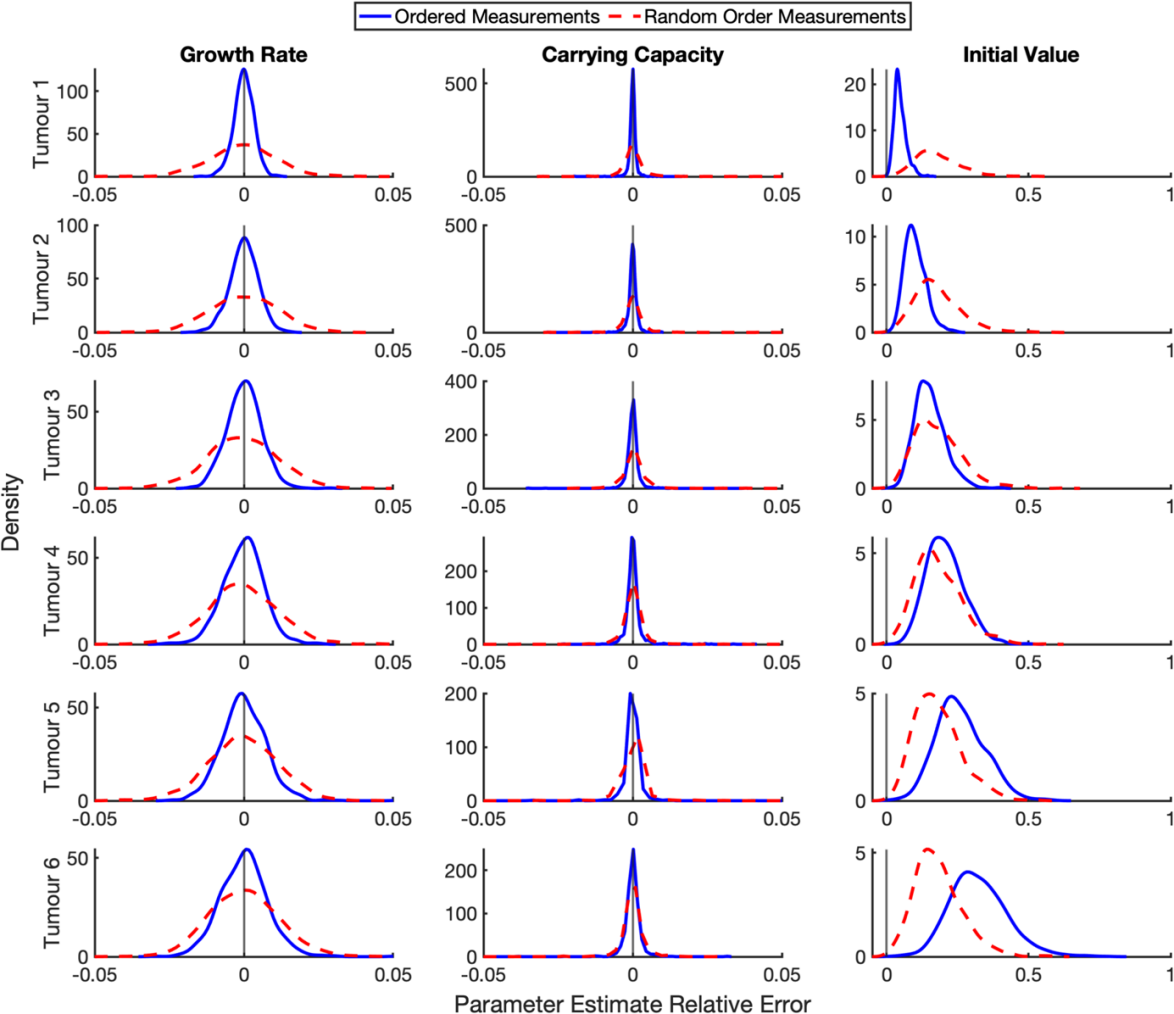
Parameter estimation for individual tumours. The density of relative error of the parameter estimates for individual tumours using least squares fitting of 10000 synthetic datasets per tumour with parameter values $y(0)\sim \mathcal {U}(100,120)$, growth rate $r\sim \mathcal {N}(0.08,0.02^2)$, and carrying capacity $K=4000$. The synthetic datasets for each of the six tumours were fitted individually for growth rate, *r*, carrying capacity, *K*, and initial value, *y*(0), are included in the fit routine. Note the different vertical scales on the plots

From these 10000 datasets, we also inferred parameters for each individual tumour, i.e., we fitted individual trajectories with only a single observation per protocol time. As each tumour and realisation of the data has distinct parameter values, we calculated the relative error of each estimate with respect to its true value (for example, the relative error in the growth rate is $(\hat{r} - r)/r$). Figure 10 presents the resulting relative error densities. It can be seen that the growth rate and carrying capacity can be reliably estimated at the individual-tumour level, but that the initial values are again over-estimates of the true values.

In growth studies, parameter inference often aims to predict the long term system behaviour – for example, estimating how large (or fast) a tumour will grow or how it might respond to treatment. This long-term behaviour is not governed by the initial value. It is therefore noteworthy that the parameters governing the long-term tumour growth can be reasonably inferred even under substantial measurement error (mean error $\mu _M = 1/6$, corresponding to an average one-day interval between the first and the last measurements). In this case, the carrying capacity is slightly underestimated but the density of the estimates is left-skewed with the highest density near the true carrying capacity.

In this synthetic data study, only the inference of the initial tumour size appears to be significantly affected by measurement error. This outcome is expected as the measurement error effectively acts as a delay on the measurements. Notably, the sequence in which measurements are taken has little influence on the inference of any of the parameters. One might expect the ordered measurements to have a greater impact on inference, given that the last tumour measured always experiences the longest delay. However, this does not appear to be the case. The parameter estimate density for all tumours is centred around the true value, although the variance increases for mice later in the measurement order. There is an inflation of the variance of the estimate when the sequence of measurements is randomly ordered rather than fixed but this variance is relatively consistent across all the tumours.

The effect of misreported times on population inference was also investigated in Wang and Davidian (1996), which recommended avoiding incorrect time recording or, at minimum, recognising and accounting for such errors when they cannot be prevented. The study found that there may be substantial bias in estimators of intra-individual parameters. On the other hand, population level inference was less affected; moderate amounts of error could degrade the quality of estimators, although inference appeared robust to small errors. In our synthetic tumour study, we do not observe any significant bias in intra-individual (individual tumour) parameter estimates. This discrepancy may reflect differences in the underlying models and error distributions used in the two studies.

***GLUT4 Translocation*** For our final investigation, we use a Bayesian parameter estimation to demonstrate that the effects of measurement error persist with different inference methods. We examine the compartmental model of GLUT4 translocation given in Yeh et al. (1995); Govers et al. (2004). The model consists of two compartments: the plasma membrane and an internal exchanger. The model is given by

16 $$\begin{aligned} \begin{aligned} \frac{dP}{dt}&= k_{ex}S - k_{en}P \\ \frac{dS}{dt}&= k_{en}P - k_{ex}S \\ \text {Total}&= P + S \end{aligned} \end{aligned}$$

where *P* is the amount of GLUT4 on the plasma membrane, *S* is the amount of GLUT4 in the internal exchanger, $k_{en}$ is the endocytosis rate, and $k_{ex}$ is the exocytosis rate. Conservation is enforced so that the total amount of GLUT4 in the system is constant. As is commonly done we take *P* and *S* to represent the proportion of GLUT4 in each compartment; in other words, the total in the system is taken to be ($P+S=1$).

The solution to this model, in the *P* compartment, is

17 $$\begin{aligned} P(t) = (P(0)-M)e^{-kt} + M, \end{aligned}$$

where $$M = {k_{ex}}/({k_{ex}+k _{en}})$$ is the carrying capacity ($$\lim _{t\rightarrow \infty }g(t) = M$$), *P*(0) is the initial condition, and $$k = k_{ex}+k_{en}$$ is the effective exocytosis rate. This is an exponential rise to a plateau. The parameter vector to be inferred is $$\theta = (P(0), k, M)$$. The set of observation protocol times is $$W = $${0, 0.5, 1, 2, 5, 10, 15, 20, 25, 30, 45, 60}.

Synthetic data is generated from the lognormal model

18 $$\begin{aligned} Y(t) \vert \theta \sim Lognormal(\mu ,\sigma ^2). \end{aligned}$$

We set the target variance $\sigma _Y^2$ and target mean $P(\theta ,t)$ on the original scale and set the log–scale parameters as

$$\begin{aligned} \mu = \ln \left( \frac{P(\theta ,t)^2}{\sqrt{P(\theta ,t)^2 + \sigma _Y^2}}\right) , \qquad \sigma ^2 = \ln \left( 1 + \frac{\sigma _Y^2}{P(\theta ,t)^2}\right) \end{aligned}$$

using the parameterisation used in Gustavsson et al. (2014); Heijungs (2024); Smith et al. (2023). It is also common to set the log-scale parameters using ‘geometric parameters’ $\mu = \ln (P(\theta ,t))$ and $\sigma = \ln (\sigma _Y)$ (Murphy et al. 2024; Limpert et al. 2001; Kreutz et al. 2007). In this alternative formulation $P(\theta ,t)$ is the median and $\sigma _Y$ is the multiplicative standard deviation and determines the shape of the distribution.

**Fig. 11:**
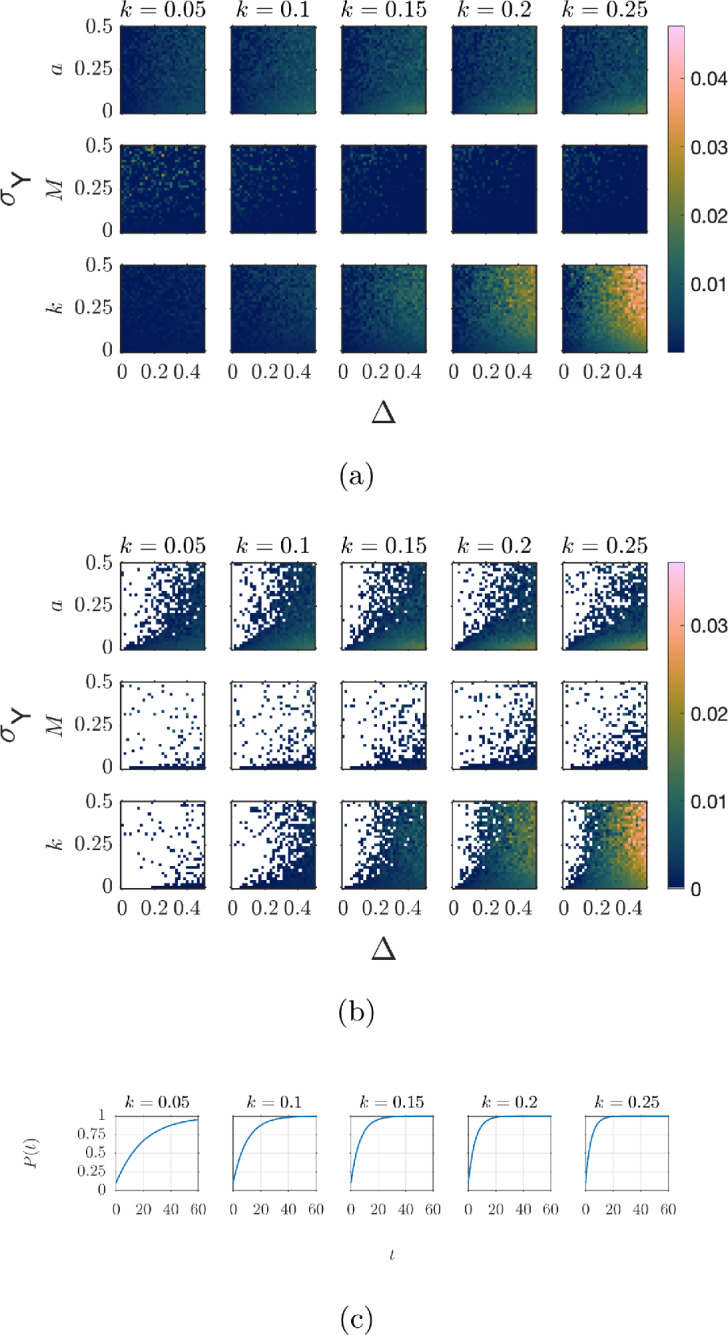
Bayesian parameter estimation metrics with the rate parameter, *k*, varied across each column and the carrying capacity and start point set at $M=1$, and $P(0)=0.1$ respectively. Rows correspond to metrics for each parameter. Each heatmap shows the metric calculated from 1000 samples at each time-point with varying $\Delta$ and $\sigma _Y$ values in the generated data. Figures indicate **(a)** the absolute error in the MAP estimate; **(b)** the distance from the true parameter value of the 95% credibility interval, white indicates the true parameter value is contained within the credibility interval; **(c)** shows the model output for the given set of parameters

Data is generated using combinations of parameter values

$$ \begin{aligned} P(0)&\in \{0.05,\,0.1,\,0.15,\,0.2,\,0.25\},\\ k&\in \{0.05,\,0.1,\,0.15,\,0.2,\,0.25\},\\ M&\in \{1,\,8,\,15,\,22,\,29\} \end{aligned} $$

as the true parameter sets with multiple replicates for each protocol time. Model outputs for each parameter set (without measurement error) are shown in Figures 11(c) 11(c). We consider two sample sizes for our synthetic data. The first, consists of 1000 repeated measurements at each time point. The second of only eight measurements per time point. The results presented in the main text correspond to the 1000-sample case, with the results for the 8-sample case given in Appendix 2.

We introduce a biased error, *U*, to the true measurement times,

$$\begin{aligned} t = W+U, \end{aligned}$$

where $U\sim \mathcal {U}(0,\Delta )$. The posterior distribution for each combination of the length of the support of the uniform distribution $\Delta$ and the target variance $\sigma _Y^2$ is approximated using an SMC sampling (see Appendix 1).

For each parameter combination, we evaluated three metrics of fit quality: the absolute error of the maximum a posteriori (MAP) estimate, the width of the 95% credibility interval, and the distance between the true parameter value and the interval, definitions for each metric are given in Appendix 1. We present these metrics in heatmaps as functions of $\Delta$ and $\sigma _Y^2$ under different true parameter values. The error in the MAP estimate and the distance to the confidence interval are presented below. Figures for the width of the confidence interval can be found in Appendix 2.

Figure 11a shows the absolute error in the MAP estimate for each of the three parameters, represented by the rows. Each heatmap varies both $\Delta$ and $\sigma _Y^2$, and the columns represent changing growth rates *k*, with carrying capacity fixed at $M=1$ and the initial condition at $P(0)=0.1$. We see that the error in MAP estimates of all parameters remain relatively consistent, except when both $\sigma _Y$ and $\Delta$ are large in combination with a large *k*. Higher *k* values – corresponding to faster growth rates – amplify the influence of errors on parameter estimates. As *k* increases, the number of observations before the system reaches steady state declines, leaving fewer data points to constrain the model during the transient phase.

Figure 11b shows the distance between the true parameter value and the credibility interval, with rows and columns having the same meaning as previously. Although the accuracy of the *k* estimate decreases with increasing error, it can be seen in Figure 11bthat the greater data heterogeneity (the larger $\sigma _Y$) is, the larger the measurement errors in time can be before the credibility intervals fail to contain the true parameters. Indeed, the widest credibility intervals occur when both $\sigma _Y$ and $\Delta$ are large, yet higher $\sigma _Y$ consistently improves containment of the true values. In contrast, with no heterogeneity ($\sigma _Y=0$) the true parameters fall outside the credibility interval even for very small timing errors. In general, the determination of the width of the credibility interval is dominated by the variance in the dependent variable measurements $\sigma _Y^2$, rather than the amount of measurement error.

**Fig. 12:**
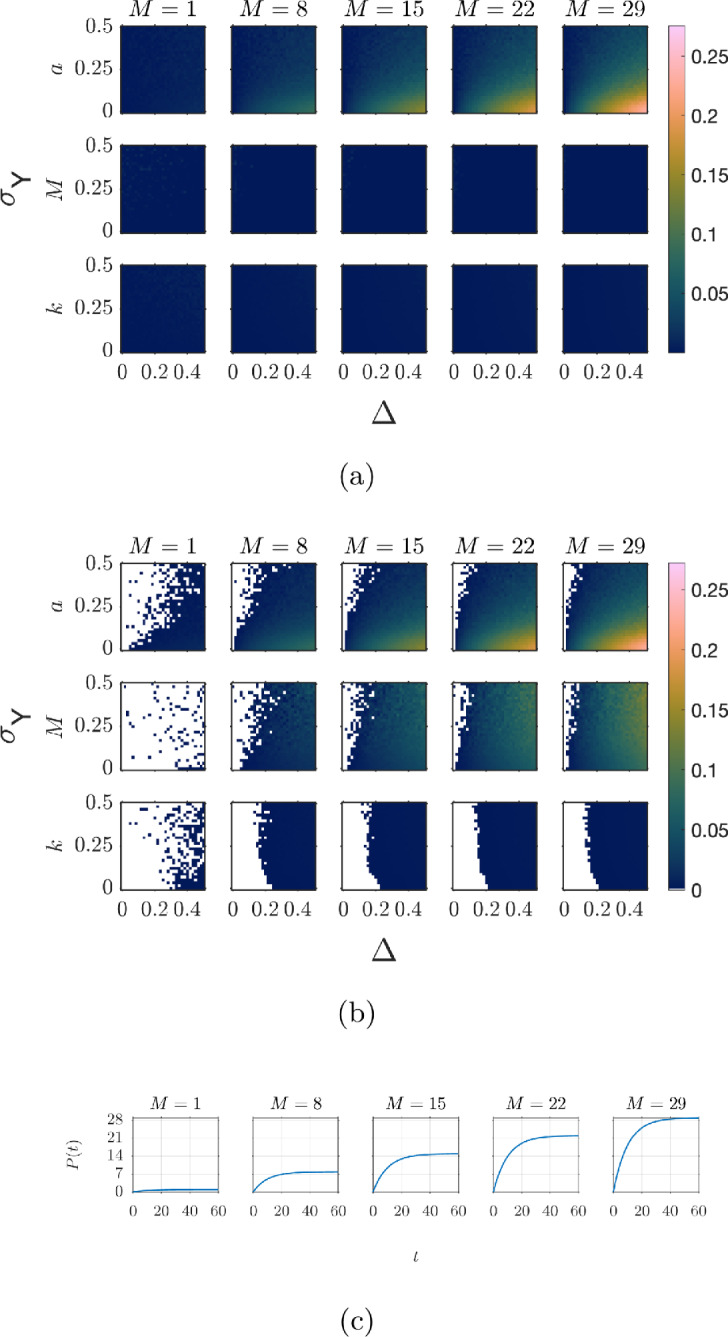
Bayesian parameter estimation metrics with the carrying capacity, *M*, varied across each column and the carrying capacity and start point set at $k=0.1$, and $P(0)=0.1$ respectively. Rows correspond to metrics for each parameter. Each heatmap shows the metric calculated from 1000 samples at each time-point with varying $\Delta$ and $\sigma _Y$ values in the generated data. Figures indicate **(a)** the absolute error in the MAP estimate; **(b)** the distance from the true parameter value of the 95% credibility interval, white indicates the true parameter value is contained within the credibility interval; **(c)** shows the model output for the given set of parameters

Figure 12a represents the MAP estimate errors and Figure 12b represents the distance between the true parameter value and the credibility interval with the carrying capacity *M* varied across columns. The growth rate is fixed at $k=0.1$ and the initial condition at $P(0)=0.1$. Changes in carrying capacity *M* have minimal impact on parameter estimates. Only the estimate of the initial value *P*(0) worsens when *M* is large in combination with high timing error and low heterogeneity ($\sigma _Y$). For large carrying capacities, the high initial rate of change makes early time points particularly susceptible to timing errors.

In all cases, except for errors in MAP estimates of *k* when *k* is large, increasing $\sigma _Y$ provides greater tolerance for timing error as may be expected. In other words, for a given acceptable error in parameter estimates, greater heterogeneity in the data $\sigma _Y$ allows a greater tolerance in timing errors.

Similar results were seen for small sample sizes with 8-measurements per time points, but with less clear trends; see Appendix 2. For the smaller sample sizes, the trends were less distinct, with the smaller sample sizes leading to more diffuse heatmaps due to a greater influence of sampling variability.

## Corrections for Measurement Error

In this section, we review methods that correct for measurement errors in inference. We highlight the requirements of each method so the reader can identify methods that may be suitable to their specific setting. First, we review methods that require additional data, either replication or validation data, to estimate the error and correct for it. Then, we present methods that do not require any additional data. Some of these methods do require assumptions on the error variance – which may be acquired via additional data or external sources. Finally, we present likelihood and Bayesian methods, which require strong distributional assumptions. These methods require the distribution of the error to be fully specified.

We focus on methods that are model agnostic. Some methods are applicable to only to linear models. These methods are generally capable of correcting parameter estimates exactly (or very close to exactly) and have analytically known behaviours. We will specify these methods as they arise. Methods that are more generally applicable are approximate corrections, even when applied to a linear case, and specialised methods should be chosen when the requirements are met.

An alternative method, that we do not explore in this text, is instrumental variable (IV) methods. These are applicable when a suitable auxiliary variable – an ‘instrument’ – is available. A valid instrument is correlated with the independent variable *X* but not with its error *U* and has no independent effect on the dependent variable *Y* (the only effect is through the correlated *X*. See (Wang and Hsiao 2007) for a method when the predictor and instrumental variables are normally distributed.

For time-series data where multiple individuals trajectories can be tracked, often giving waveforms or signals, a popular method is time-warping (Krotov et al. 2025; Srivastava and Klassen 2016; Cavill et al. 2013). Given a set of signals, time-warping finds an optimal alignment between the signals – such as by aligning peaks and troughs across different signals. This method has been found to handle variability in both spatial and temporal domains (Krotov et al. 2025). These methods, however, are not further explored in this work.

Our aim in this paper is to identify methods that are applicable to different modelling scenarios in the biological sciences. Implementations for some of the methods discussed can be found in Buonaccorsi (2010); Carroll (2006); Buzas et al. (2004); Fuller (1987). For reviews on methods with a focus on applications in economics see (Schennach 2016; Chen et al. 2011); robustness to model misspecification is reviewed in Guolo (2008), while applications to nutritional epidemiology are reviewed in Keogh and White (2014).

### The Use of Additional Data

In some studies, additional data can be collected to aid in estimating and correcting for measurement error. Here, we outline three main types of additional data: internal replication data, external replication data, and internal validation data. We outline each type, noting restrictions and challenges relevant to the biological science before reviewing methods that make use of additional data. These methods are useful as the additional data provides a means to estimate the measurement error. However, this data is rarely available in practice and the sampling distribution of the resulting estimate is highly skewed in small samples (Carroll 2006).

Internal replication data are replicate measurements from within the same experimental sample. For example, the *j*th observation of the *i*th individual, $W_{ij}$, might be measured multiple times such that we have observations $W_{ijk}$, the *k*th replicate for the *ij*th observation. Note, that these are technical replicates not biological (or experimental) replicates. Biological replicates capture information about biological variation – they are samples from distinct sources, such as different patients, animals, or cells cultured separately. Technical replicates are repeated measurements of the same sample with low biological variation. For example, the same samples being run through an assay multiple times or, for in vitro cell culture, samples from the same culture in different wells of the same plate are technical replicates (Chan and Teo 2020).

External replication data are replicate measurements taken from samples outside of the main study. An example is calibrating a measuring device using a known quantity to understand the variability in the device. External replicate data may not always be transportable to the study of interest as the variance of the error may not be preserved across samples (Buonaccorsi 2010; Carroll 2006). Both types of replicate data are usually used for estimating the variance of the error term.

Internal validation data refers to the case where the true (error free) values *X* for mismeasured variables are observed for a subset of observations. For example, the bulk of measurements may be taken with a cheap, readily available but less accurate test while a subset is taken with a more costly gold standard test, as in the earlier example in Section 2 where the more accurate refined western blot procedure was used on only a subset of samples when testing for HSV-2 (Carroll et al. 1993). The main limitation on the use of validation data is that it can be costly to obtain.

Below, we present three methods that make use of additional data to correct for measurement errors. The first only requires two replicate measurements for each observation, however, has strong requirements for the form of the model. Then, we present a method that requires validation data. The final method, regression calibration can make sue of any type of additional data that is usually applied to linear models but has extensions to other classes of models.

***A Method for Partially Linear Models with Longitudinal Data*** A method that makes use of replicate measurements applicable to linear marginal and partially linear models is proposed in Qin et al. (2016). It is assumed that there are two replicate measurements for $X_{ij}$ given by $W_{ij(1)} = X_{ij}+U_{ij(1)}$ and $W_{ij(2)} = X_{ij}+U_{ij(2)}$, where the measurement errors $U_{ij}(1)$ and $U_{ij(2)}$ are independent. The reader can see (He et al. 2005) for details on calculations of the method and Lin et al. (2018); Yi et al. (2012) for extensions to dropout data when a subject is removed from the study prior to completion of all measurements. If replicate measurements are available, see (Qin et al. 2016) for a proposed robust estimation method for longitudinal data with missing data and measurement error.

***A Method for Using Validation Data*** The use of validation data is explored in Sepanski and Carroll (1993). Here, in addition to the primary dataset (consisting of (*W*, *Y*)), a validation dataset is given where both *X* and *W* are observed. The validation dataset can contain (*X*, *W*, *Y*) or only (*X*, *W*) observations. Due to the use of validation data, no assumptions are made on the measurement error. The technique only requires that the first and second moments of *Y* given *X* be specified. This is reasonable when better equipment is available for some measurements. For example, more powerful imaging equipment may be available for a subset of subjects – for example more powerful MRI machines can provide better spatial resolution in images but are not widely available (Bates et al. 2023). However, obtaining validation data may be difficult if the error is inherent to the experimental protocol, e.g., if putting cells on ice does not immediately stop movement in the cell and we cannot ascertain the actual time the cells have been reacting to an agent.

***Regression Calibration*** Regression calibration replaces *X* with the expectation of *X* given the observed data (*W*, *Z*), (i.e., $\mathbb {E}[X\vert W,Z]$) where *Z* are independent variables measured without error (Prentice 1982; Carroll 2006). The estimation of *X* is done via a model

$$\begin{aligned} \mathbb {E}[X\vert W,Z] = m_X(W,Z,\gamma ) \end{aligned}$$

with model parameters $\gamma$. This is called the calibration model and is used as a replacement for *X* once the model parameters are estimated using the observed data. Once the replacement is made, the usual inference methods can be used as if *X* were observed (Pierce and Kellerer 2004).

The method is most useful for generalised linear models where the approximation is either exact, or very close to exact (Carroll 2006). Methods for a linear approximation to the calibration function are given in Carroll (2006). A method using James-Stein estimates for additive error models with replicate data and that requires only *W* (*Z* is not required) is given in Whittemore (1989). The method is extended to logistic models in Rosner et al. (1989) and a general class of models in Carroll and Stefanski (1990). These methods require additional data, either replicate observations or validation data and assume classical error. Note, that for an unbiased Berkson error we have that $\mathbb {E}[X\vert W] = W$, so no extra information is obtained.

Regression calibration methods do not work well with highly nonlinear models. However, if the problem can be written as a quasi-likelihood and variance function (QVF) model then an expanded regression calibration method can be applied. QVF models require models for the mean and variance of the data, $\mathbb {E}[Y\vert X]$ and $\textrm{Var}[Y \vert X]$ respectively. This method is most useful when the error model is Berkson as the method does not require validation data. We direct the reader to Carroll (2006) for further information.

### Methods Requiring Assumptions on the Error Model

In this section, we present methods that do not require additional data, but rather require some knowledge about the variance of the measurement error. In some cases, the variance may be estimated via the use of additional data – but the methods themselves do not specify how the variance is calculated.

We first present two methods (reliability ratio and Fuller’s method of moments) that apply to linear models when the measurement error variance $\sigma _u^2$ is known. Then, two model agnostic methods are presented: orthogonal regression and simulation extrapolation. That is, they do not have requirements about the form of the model. The first of these requires knowledge only of the measurement error variance $\sigma _\epsilon ^2 / \sigma _u^2$. The final method presented requires the variance $\sigma _u^2$ to be estimated and using model simulations to learn the effect of the measurement error and extrapolate to a zero error case.

***Reliability Ratio*** If the measurement error variance $\sigma _u^2$ is known then the reliability ratio (Equation (7)) can be used to correct parameter estimates in linear models. Replicate measurements or validation data allow estimation of $\sigma _u^2$, from which the reliability ratio $\lambda$ can be inferred and the parameter estimate corrected. The main advantage of this approach is its simplicity and that it provides an exact parameter estimate for classical additive error in linear models, provided $\sigma _u^2$ is known. However, the limitation is that it relies on an accurate value for $\sigma _u^2$; if this variance is misspecified, the correction does not correctly account for the bias in the estimate. It is also not applicable to nonlinear models.

***Fuller’s Method of Moments*** Fuller’s *method of moments* (Fuller 1987) is an easy to implement method when the measurement error variance $\sigma _u^2$ is known. This method is applicable only to linear models with additive measurement error. As with the reliability ratio, the main limitation of this method is the capacity to know the variance of the error. The measurement error variance cannot always be known but can be estimated, typically from additional data. The estimated variance cannot be used directly in the method as this results in the standard errors of the parameter estimates being underestimated (Meijer et al. 2021). Further, Meijer et al. (2021) presents a method to correct the errors of the estimator in this case, extends the approach to panel data (also known as longitudinal or cross-sectional time-series data), and provides an overview of the effects of measurement error on *t*-statistics and $R^2$ values.

***Orthogonal Regression*** *Orthogonal regression* (Fuller 1987; Carroll 2006) – sometimes referred to as the *linear statistical relationship* (Tan and Iglewicz 1999) or *orthogonal least squares* – is a simple to implement method to correct for measurement error that does not require any additional data or assumptions about the error. Orthogonal regression minimises the orthogonal distance between the observed data $\{(w_i,y_i)\}_{i=1,\dots , N}$ and the model output ${y}(\theta ,x)$, weighted by $\eta$. The parameter estimate is given by the $\theta$ that minimises

19 $$\begin{aligned} \min _{(\theta ,x)} \sum _{i=1}^N \left( \left( {y}_i - y(\theta ,x_i)\right) ^2 + \eta \left( {w}_i-x_i\right) ^2\right) \end{aligned}$$

over the unknown parameters $\left( \theta ,x_1,\dots ,x_N\right) .$ The weighting $\eta$ requires knowledge of the ratio of the measurement error variance, i.e., $\eta = \sigma _\epsilon ^2 / \sigma _u^2.$ Some sources, for example (Casella and Berger 2002), define orthogonal regression only for the special case $\eta = 1$, in which the minimisation reduces to the sum of squared orthogonal distances. Some texts advise against the use of orthogonal regression because the method does not account for errors in the equation (the variation about the fitted model of the true response without measurement error) resulting in an overcorrection of the measurement error (Carroll and Ruppert 1996). Indeed, numerical studies show that the method (with $\eta =1$) does not consistently correct the parameter estimates. The correction appears to depend on the scale of the data; see Appendix 2 for an illustration.

Orthogonal regression, with $\eta =1$ was compared to three other estimation methods in Valsami et al. (2000). The Hill equation was used to model drug binding as a synthetic data study with measurement error in both independent and dependent variables and different methods were implemented to estimate the unknown parameters of the Hill equation. The four methods implemented were: geometric mean functional relationship (GMFR) (Barker et al. 1988), the maximum likelihood estimate (MLE), the perpendicular least-squares (PLS) (Ko et al. 1997) (referred to as orthogonal least squares in other texts) and the nonlinear weighted least squares. None of these methods explicitly model the measurement error and instead implement various distances from a data point to the model output. It was found that all methods performed poorly when measurement errors of the two variables were correlated. All methods were reported to perform equally well (in terms of root mean square error) with uncorrelated errors.

***Simulation Extrapolation (SIMEX)*** Simulation extrapolation (SIMEX) (Cook and Stefanski 1994; Stefanski and Cook 1995; Carroll and Ruppert 1996) is a flexible, simulation-based approach for reducing the effects of measurement error when the measurement error variance $\sigma _u^2$ is known or can be estimated. SIMEX is ideally suited to problems with additive measurement error. SIMEX has been applied to linear, non-linear, and mixed-effects models (Carroll 2006).

The core idea is to learn how parameter estimates change as the measurement error variance is artificially inflated, then use this relationship to extrapolate back to the ‘no-error’ case. In doing so, SIMEX provides adjusted estimates that aim to approximate those that would have been obtained had the independent variable been measured without error. The structure of the method is given in Algorithm 1.

**Algorithm 1:**
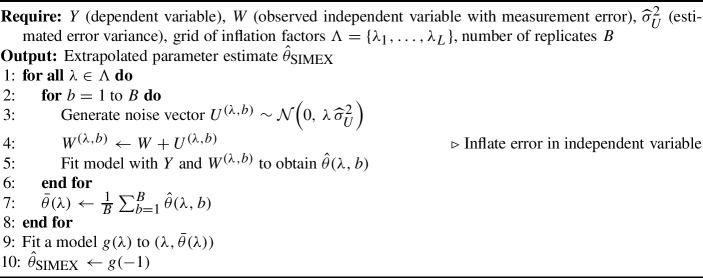
SIMEX procedure for measurement error in the independent variable with classical error $W = X + U$, where *U* is normally distributed

The main advantage of the SIMEX method is its broad applicability. It is model agnostic and does not require any analytic model solutions, making it appealing to those who work with computational models. However, as with previous methods, the ability to correct parameter estimates using SIMEX is limited by how well the variance of the measurement error $\sigma _u^2$ can be estimated and the correct specification of the error model. Depending on the model, it can be more computationally expensive than alternative methods due to repeated parameter estimation.

### Likelihood and Bayesian Methods

Previous methods require few, if any, assumptions on the distribution of *X* or *W*. The likelihood methods that we present in this section require distributions to be fully specified. However, they can generally be applied to a larger class of problems. The previous methods each required a specific model structure, extra data, or knowledge of the measurement area variance. The following likelihood methods can also be applied to all of these cases and generally results in smaller standard errors compared to the previous methods. However, this is at the cost of additional modelling assumptions (Carroll 2006).

In this section we outline the steps to extend the construction of a likelihood function to account for measurement error in the independent variable. The method requires four steps. First, the likelihood is constructed in the usual manner as if there were no measurement error and *X* is observable. Then, the measurement error model is selected. Third, the likelihood is formed to include the selected measurement error model. Finally, the likelihood is maximised or used in a Bayesian setup to estimate posterior distributions to provide parameter estimates.

**Fig. 13:**
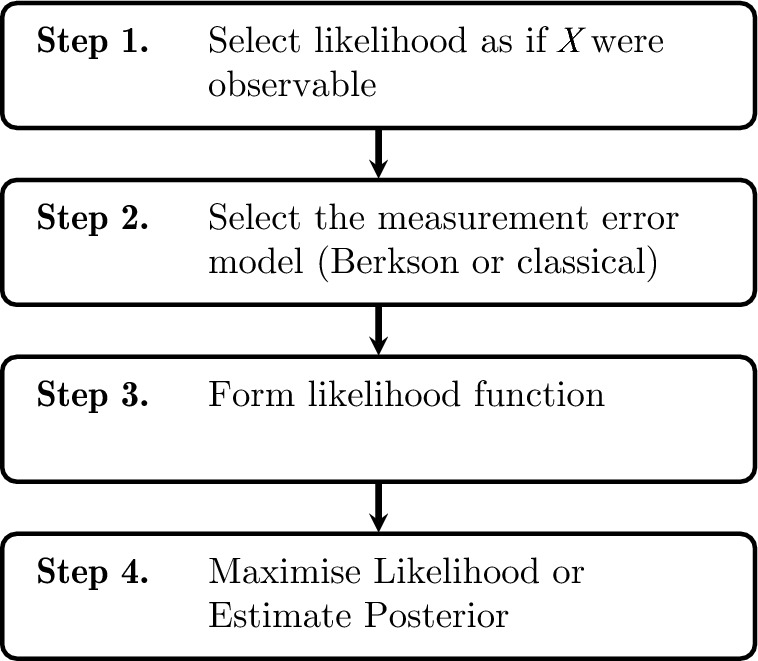
Workflow for likelihood methods (Carroll 2006)

***Step 1: The Usual Likelihood*** To build a likelihood function with measurement error, first construct the usual likelihood as if the data were free from measurement error. We have described this construction in Section 3.1. The reader can find further suggestions on likelihood construction in Murphy et al. (2024); Gershenfeld and Gershenfeld (1999).

***Steps 2 and 3: Error Model Selection and Likelihood formation*** To model the measurement error, a choice of error model must be made. In this case, rather than observing *X*, we only have observations of the error prone *W*. So, we seek the probability of seeing data *y* given an observation *w*. Generally, the first choice is whether the error is classical or Berkson. We will only briefly mention the classical case here in order to focus on the Berkson error model, which is most common for the biological sciences, i.e., experiments are typically designed as controlled experiments. Here, recalling notation from Section 3.1, we specify a density for the measurement error model as $\phi (\cdot )$.

For a classical error model, we need to specify a model for the observed values *W* given the true values *X*, that is, $\phi _{W\vert X}(\cdot )$, and a model for *X* (the distribution of the true *X* values). Given these densities, we have

20 $$\begin{aligned} \phi _{Y\vert W} = \int _x \phi _{Y\vert X}(y;y(\theta ,x),\theta )\phi _{W \vert X}(w;x,\cdot )\phi _X(x;\cdot ) \,dx. \end{aligned}$$

This formulation allows for non iid realisations of $X_i$ and $Y_i$. That is, each data pair $(x_i,y_i)$ can come from a different distribution. We do not subscript the random variables for notation clarity.

The Berkson error model requires specifying a model for the true values given the observed values (*X* given *W*). Note that the distribution of *W* does not convey information about the parameters of interest $\theta$ and is not explicitly written in the likelihood (Carroll 2006). It is easy to see why the controlled experiment case does not give information where the observed values *W* are predetermined. Unlike the classical error model, a density for the true *X* values is not required. For Berkson error, we have

21 $$\begin{aligned} \phi _{Y \vert W} = \int _x \phi _{Y\vert X}(y;y(\theta ,x),\theta ,\cdot )\phi _{X \vert W}(x;w,\cdot ) \,dx. \end{aligned}$$

For both error cases, the likelihood for the problem is the product over the entire sample (as in Equation (3)) with the appropriately chosen $\phi _{Y \vert W}$. That is

$$\begin{aligned} \mathcal {L} (\theta \vert y) = \prod _{i=1}^N \phi _{Y \vert W}(y_i;w_i,\theta ), \end{aligned}$$

resulting in a product over the number of data points where each term is an integral. We note also that the number of parameters in the likelihood increases as it is common to include the distribution parameters in the maximisation of the likelihood or estimation of the posteriors. That is, $\theta$ now includes the distributional parameters of $\phi _{X \vert W}$ in the Berkson case. This leads to an increase in computational complexity compared to the likelihood without measurement error. See (Buonaccorsi 2010) for a number of likelihood formulations for various error structures, including conditioning predictors measured with error on any predictors measured without error. An example likelihood with normally distributed errors is constructed in Appendix 3.

***Step 4: Maximisation of Likelihood or Estimation of Posterior*** The construction of the likelihood to include the measurement error then allows the usual frequentist (MLE) and Bayesian approaches to parameter estimation to be used. That is, to estimate parameters the likelihood is computed and either maximised or used in a Bayesian framework to estimate posterior distributions; as discussed in Section 3.1. The key difference is the added computational complexity introduced by the product of integrals. We provide some references below to methods’ studies that estimate these likelihood functions in a variety of contexts; these are also summarised in Table 8.

In Rabe-Hesketh et al. (2003) a model for the risk of disease given exposure is considered. Non-parametric methods for the calculation of the MLE are given for measurements with skew distributions. It is noted that MLE estimates are consistent if data are missing at random, i.e., the probability of a value being missing does not depend on the missing value itself.

Semi-parametric approaches are given in Schafer (2001); Aitkin and Rocci (2002) where no distributional assumptions on $\phi _X(x)$, the true independent variable values, are required. Computational implementations are provided with examples for applications. Note that this is only useful for the likelihood given in (20), as the likelihood for Berkson error does not require the distribution of *X*. Adaptations to the likelihood function when additional information is available – extra information including internal replication data, internal validation data, and external estimates of measurement error – are given in Schafer (2001). However, the use of these methods was dependent on sample sizes. For the use of internal validation data, if the number of exact observations is large, the computations may become intractable.

Frequentist and Bayesian methods are used to estimate parameters for a generalised linear mixed measurement error model (GLMM) in Torabi (2013). GLMMs are applicable when data is expected to be correlated within groups. For example, clustered data (patients sampled from within doctors – patients with the same doctor are expected to be more similar) or longitudinal data (repeated measurements from the same subject – pre- and post-treatment measurements or a drug concentration over time within a single patient). Likelihood-based inference for longitudinal data has also been implemented in Zhou et al. (2024).

**Table 8:** Examples of likelihood-based estimation methods in the presence of measurement error

Method	Data Type Considerations	Reference
Non-parametric MLE for skewed measurements	Consistent if data are missing at random	(Rabe-Hesketh et al. 2003)
Semi-parametric methods	No assumption on $\phi _X(x)$; applicable to classical likelihood but not Berkson	(Schafer 2001; Aitkin and Rocci 2002)
Likelihood adaptations with supplementary information	Works with internal replication, internal validation, or external error estimates; large validation sets may be computationally intensive	(Schafer 2001)
Frequentist and Bayesian estimation for GLMMs	Suitable for clustered and longitudinal data	(Torabi 2013)
Likelihood-based inference	Focus on longitudinal data	(Zhou et al. 2024)

**Fig. 14:**
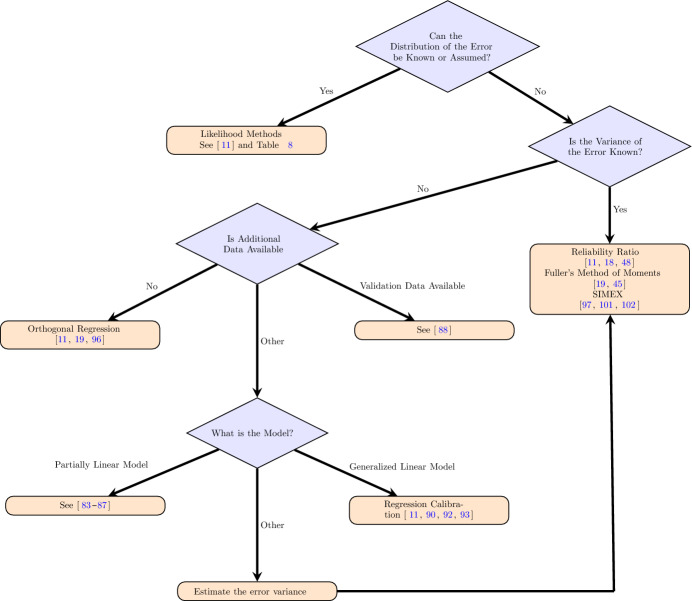
Decision tree for the selection of method to correct for measurement error

A range of methods have been reviewed in this section. Each method has its own requirements and assumptions for use. A decision tree is given in Figure 14 to assist modellers in finding the method best suited to their scenario. The tree directs readers to both the relevant methods and literature.

## Discussion

Measurement error is an unavoidable feature of experimental data collection. Whether due to limitations of equipment or human error, there is almost surely some level of error present in the data that mathematical modellers rely on to draw conclusions. This naturally raises two questions: To what extent do such errors distort the outcomes of inference, and what strategies are available to mitigate their impact?

Mathematical modellers are well equipped to address measurement error in dependent variables, and have a vast toolkit for handling these errors. However, they may not be as aware of measurement error for the independent variable – especially when their data is collected from a controlled experiment. This study examines the consequences of ignoring such errors during inference and reviews correction methods from the statistical literature. By providing context of statistical methods, we aim to expand the modelling toolkit and support more robust inference and experimental design.

Naive inference in the presence of measurement error can yield biased parameter estimates, with the magnitude and direction of bias depending on the model, the error structure (Berkson or classical), and the experimental design (controlled or non-controlled). In linear models, substantial bias in slope estimation arises only under classical error in non-controlled experiments. In contrast, the amplitude parameter of the oscillatory model was consistently underestimated across all error and experiment types in our numerical experiments. This appears to result from measurement error compressing the observed data range. More broadly, we expect that models with pronounced turning points will exhibit systematic bias in parameter estimates. For example, systems that exhibit an overshoot may see data biased away from that point, as the error distorts the curvature of the observed trajectory.

To investigate the impact of measurement error in nonlinear systems, we examined three growth models commonly used in biological applications: parasite growth, tumour growth, and exponential growth to a plateau. These models were deliberately selected for their simplicity, to mitigate against any observed bias being an artefact of model complexity. Each investigation used distinct experimental protocols and distinct error models including biased and unbiased errors and correlated and uncorrelated errors. We see that measurement error associated with the independent variable is the primary driver of bias in parameter estimates, not the error associated with the dependent variable. However, the magnitude of error required to induce appreciable bias in these models exceeded levels typically encountered in practice.

In both the parasite growth and tumour growth scenarios, the level of measurement error required to induce noticeable bias in parameter estimates exceeded that associated with measurements taken at any point in a 24 hour period. In other words, total inconsistency in measurement timing did not impact the recovery of parameters. We highlight these two cases as they illustrate distinct experimental protocols – one involving a single daily measurement, the other involving multiple measurements per day. A similarly extreme error threshold was observed in the GLUT4 translocation scenario, where the required timing error far surpassed any plausible human error. For even moderate bias in parameter estimates to arise, an experimentalist would need to mistime the insulin dose by at least ten seconds.

In our the tumour growth setting, the nature of the measurement error itself had minimal impact on parameter estimation. This is somewhat counter-intuitive: under a fixed measurement sequence, the final tumour recorded consistently experiences the longest delay, which might be expected to amplify error effects. Yet, no systematic bias was observed. The only discernible consequence was increased variance in individual tumour parameter estimates when measurement order was randomised rather than fixed, suggesting that measurement order influences precision but not accuracy.

As noted earlier, the results in Wang and Davidian (1996) indicated that intra-individual parameter estimates were more sensitive to measurement error than population-level inference. While the models and error structures in that study differ from those examined here, both share the feature of measurement times deviating from protocol times. The authors of Wang and Davidian (1996) identified that complex interplay between nonlinearity of the model and the intra-individual variance structure produces a situation where a modest measurement error results in severe biases. This indicates that the effects of measurement error in nonlinear models are complex, context-dependent and cannot be generalised across scenarios. Therefore, it is necessary to assess potential bias on a case-by-case basis, taking into account both the experimental design and the model structure.

In some cases, applying a logarithmic transformation converts multiplicative errors into additive ones, and fitting regression models to log-transformed data is a common strategy to mitigate the impact of the errors on regression (Carroll 2006). However, there is an absence of data analysis to make a strong suggestion whether to transform the data or not. We are of the view that if a transform is applied to data, the implications of the transform for the errors should be considered. Transforming the data also transforms the errors, and modellers should be aware of the assumptions they are implicitly adopting in the transformed space.

This paper focuses exclusively on additive measurement error models and does not consider multiplicative measurement errors. Multiplicative measurement error, typically written as $W=XU$, appears throughout the literature – see, for example, Pierce et al. (1992); Lyles and Kupper (1997); Hwang (1986) – though this line of research has received relatively less attention compared to additive measurement errors. Several correction methods for multiplicative error are reviewed in Lyles and Kupper (1997). A hybrid Berkson error model combining additive and multiplicative components, $W = XU_1 + U_2$, with $U_1$ and $U_2$ being independent errors, is proposed in Stram and Kopecky (2003) and applied to the Hanford Thyroid Disease Study. In that study, dose replications per individual were used to estimate shared multiplicative error and construct confidence intervals for simple models.

Measurement error has the potential to distort parameter estimates and consequently influence the conclusions drawn about system behaviour. To aid modellers in reducing these effects, when they deem it necessary, we have compiled a series of correction methods that can reduce the impact of measurement error. Each method presented requires different assumptions and available data.

Each method has its own drawbacks, and these should be carefully considered when applying them to biological data. Some of the methods presented require additional data. Therefore, whenever possible, experimental design should account for collecting some of the extra data discussed in Section 4.2. While many controlled experiments include replicate measurements, sample sizes in biological studies–such as tumour growth assays or biochemical protocols–are often small, and the number of replicates limited by cost or experimental constraints. The methods outlined require estimation of the measurement error variance from the replicate data, and with small sample sizes these estimates are often skewed. Indeed, care also needs to be taken not to conflate measurement variance and biological variability. For instance, replicates in cell assays are typically drawn from separate wells containing different cells, unlike survey studies where repeated measurements involve the same individual. This introduces biological heterogeneity and batch effects that must be accounted for Leek et al. (2010).

Likelihood-based methods avoid the drawback of requiring explicit estimates of the measurement error variance. However, they rely on full specification of the measurement error distribution, which can be difficult to justify in practice. Further work is needed to understand how such distributions can be reliably specified in biological experiments.

Likelihood-based approaches also introduce additional parameters into the inference process. Using the usual likelihood or Bayesian framework, it is common to include the hyper-parameters (the parameters from the density function) to the inference as well as the parameters from the model itself. When incorporating measurement error, the likelihood must also account for hyper-parameters from the error distribution. Unless these parameters are known *a priori*, they will also need to be estimated. This raises further questions about parameter identifiability and over-fitting. Mechanistic models often have many parameters before considering adding parameters for measurement error. Introducing further parameters without strong justification risks inflating model complexity and producing misleading conclusions.

## Conclusion

In many modelling scenarios, mistimed data may have negligible impact on inference. However, there are scenarios, such as oscillating models, where there are ramifications of ignoring measurement error. Modellers should exercise caution when applying data transformations, as these can amplify or distort underlying measurement errors–potentially leading to biased estimates or misleading conclusions.

We encourage the mathematical modelling community to assess the sensitivity of their models to measurement errors and consider what forms of error may be present in their data. Integrating measurement error correction into the modelling workflow could help mitigate the impacts of measurement error.

## Figures and Tables

**Fig. 15:**
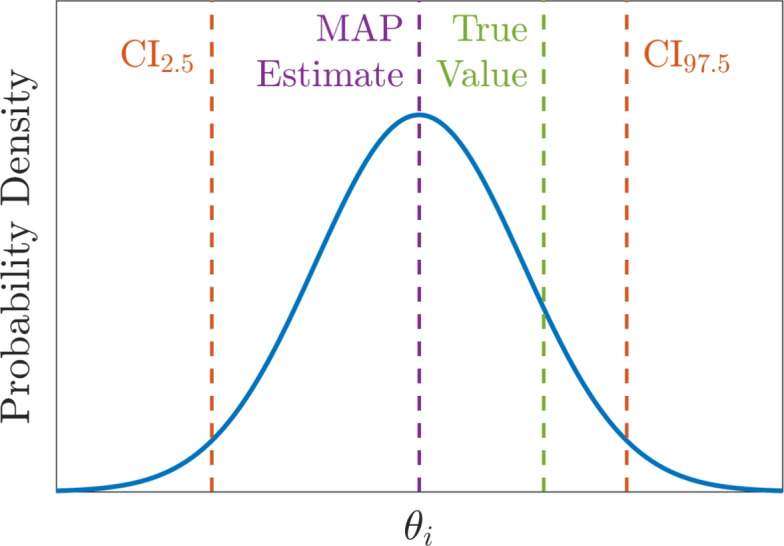
Illustration of the metrics used in this study to assess posterior distribution estimations

**Fig. 16:**
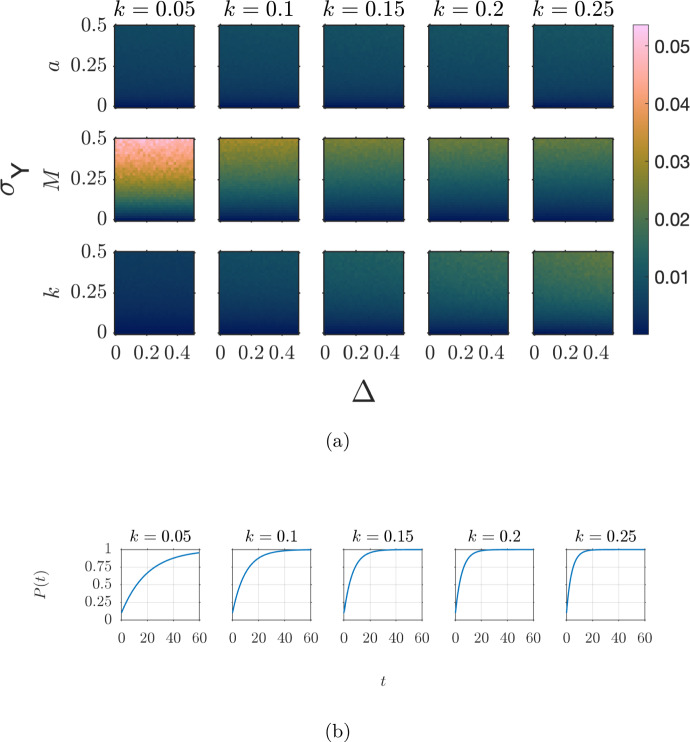
**(a)** The width of the 95% credibility interval with the rate parameter, *k*, varied across each column and the carrying capacity and start point set at $M=1$, and $a=0.1$ respectively. Each row corresponds to the CI of a different inference parameter. Each heatmap shows the width of the CI from 1000 samples at each time-point with varying $\Delta$ and $\sigma _Y$ values in the generated data. **(b)** shows the model output for the given set of parameters

**Fig. 17:**
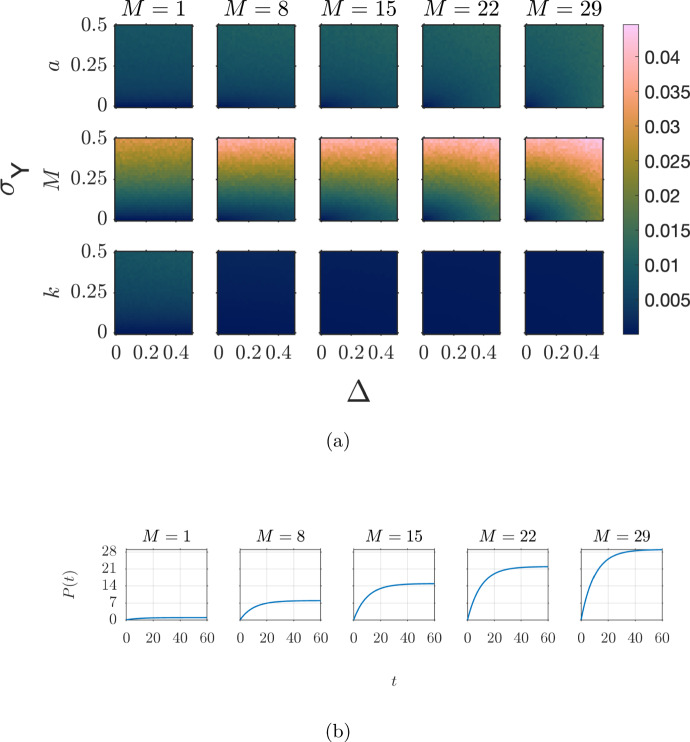
**(a)** The width of the 95% credibility interval with the rate parameter, *k*, varied across each column and the carrying capacity and start point set at $M=1$, and $a=0.1$ respectively. Each row corresponds to the CI of a different inference parameter. Each heatmap shows the width of the CI from 1000 samples at each time-point with varying $\Delta$ and $\sigma _Y$ values in the generated data. **(b)** shows the model output for the given set of parameters

**Fig. 18:**
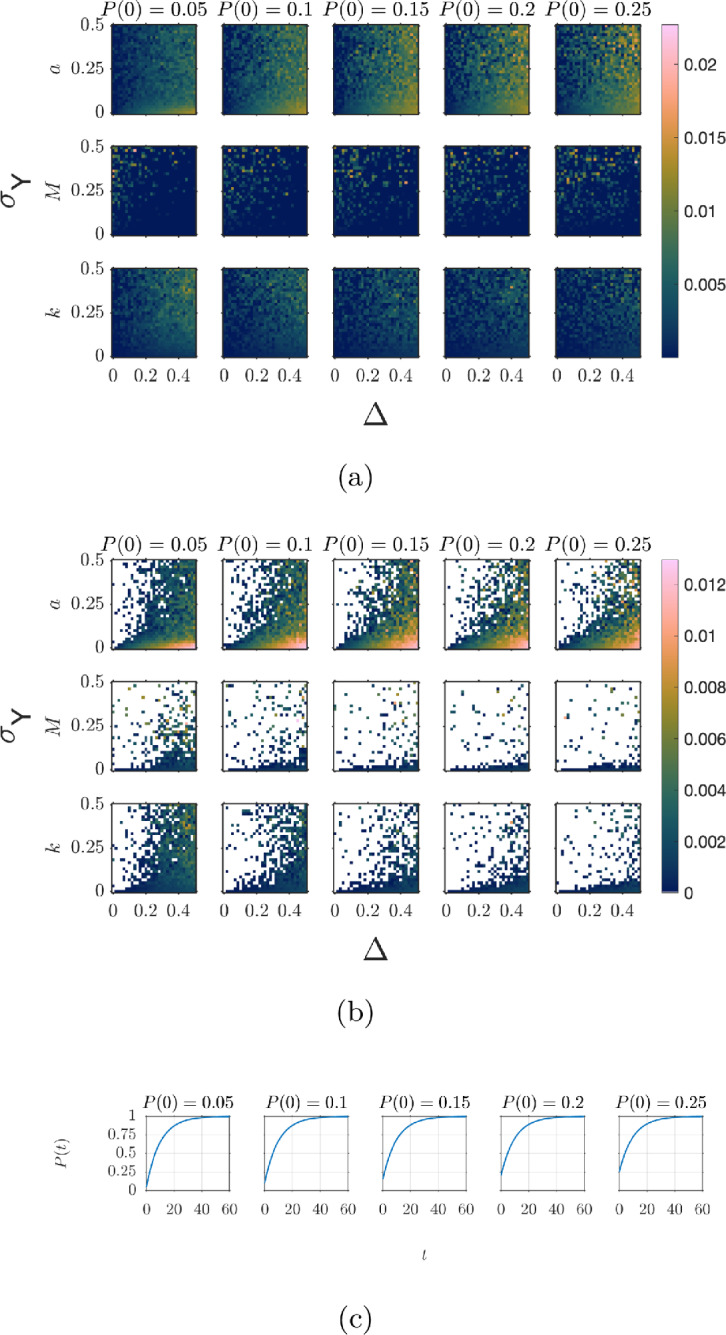
Bayesian parameter estimation metrics with the initial condition, *P*(0), varied across each column and the carrying capacity and start point set at $M=1$, and $k=0.1$ respectively. Rows correspond to metrics for each parameter. Each heatmap shows the metric calculated from 1000 samples at each time-point with varying $\Delta$ and $\sigma _Y$ values in the generated data. Figures indicate **(a)** the absolute error in the MAP estimate; **(b)** the distance from the true parameter value of the 95% credibility interval, white indicates the true parameter value is contained within the credibility interval; **(c)** shows the model output for the given set of parameters

**Fig. 19:**
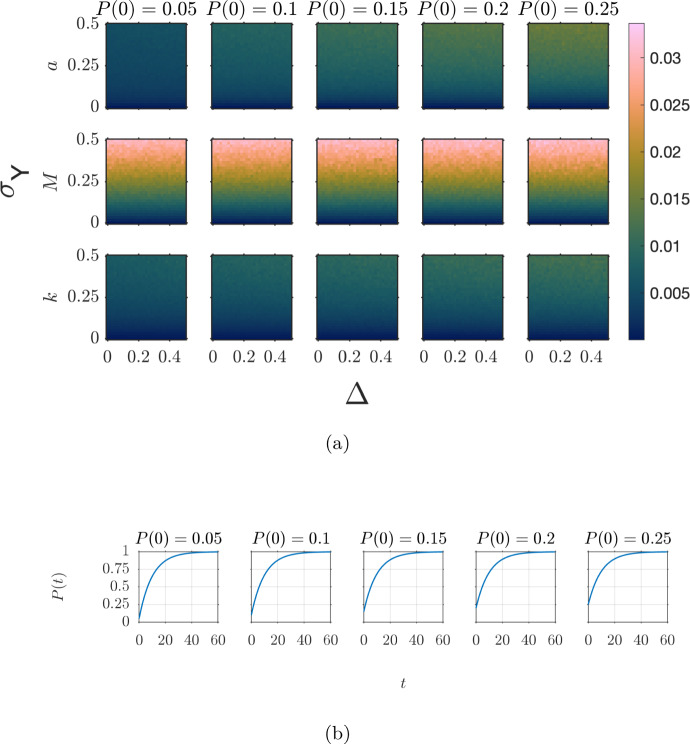
**(a)** The width of the 95% credibility interval with the initial condition, *P*(0), varied across each column and the carrying capacity and start point set at $M=1$, and $k=0.1$, respectively. Each row corresponds to the CI of a different inference parameter. Each heatmap shows the width of the CI from 1000 samples at each time-point with varying $\Delta$ and $\sigma _Y$ values in the generated data. **(b)** shows the model output for the given set of parameters

**Fig. 20:**
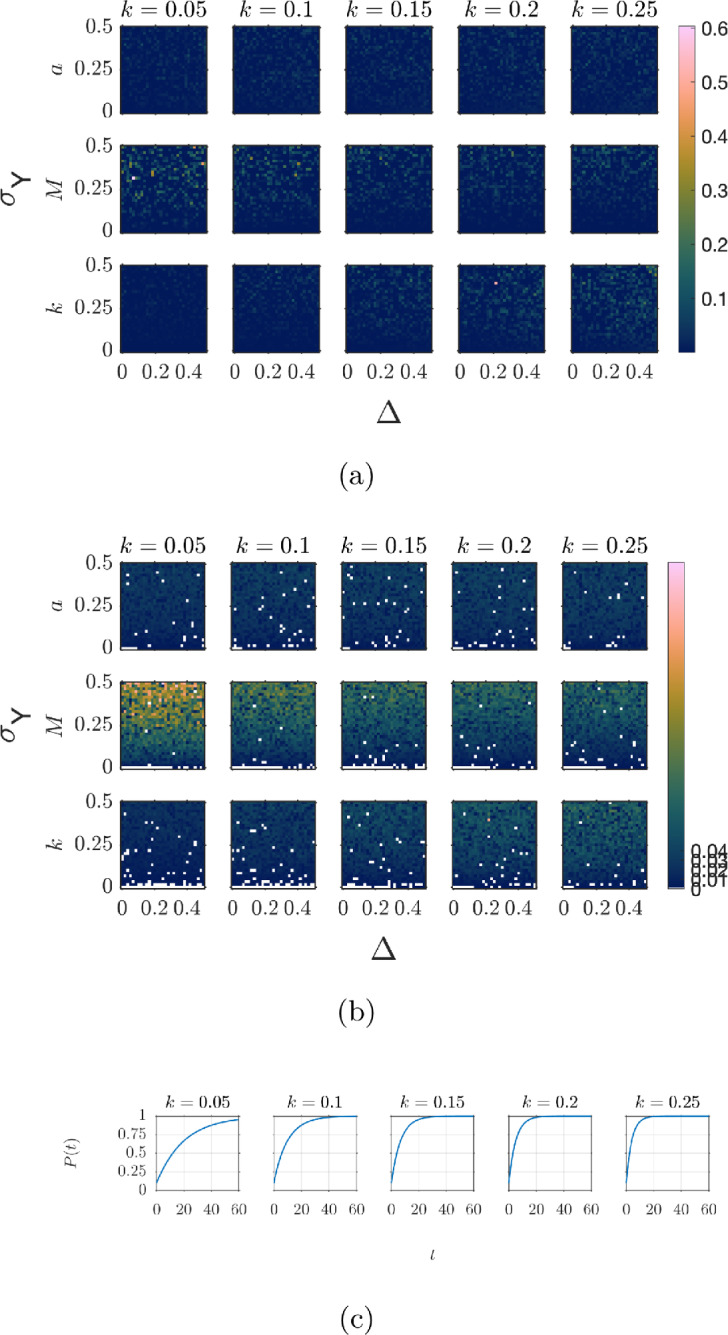
Bayesian parameter estimation metrics with the rate parameter, *k*, varied across each column and the carrying capacity and start point set at $M=1$, and $P(0)=0.1$ respectively. Rows correspond to metrics for each parameter. Each heatmap shows the metric calculated from 8 samples at each time-point with varying $\Delta$ and $\sigma _Y$ values in the generated data. Figures indicate **(a)** the absolute error in the MAP estimate; **(b)** the distance from the true parameter value of the 95% credibility interval, white indicates the true parameter value is contained within the credibility interval; **(c)** shows the model output for the given set of parameters

**Fig. 21:**
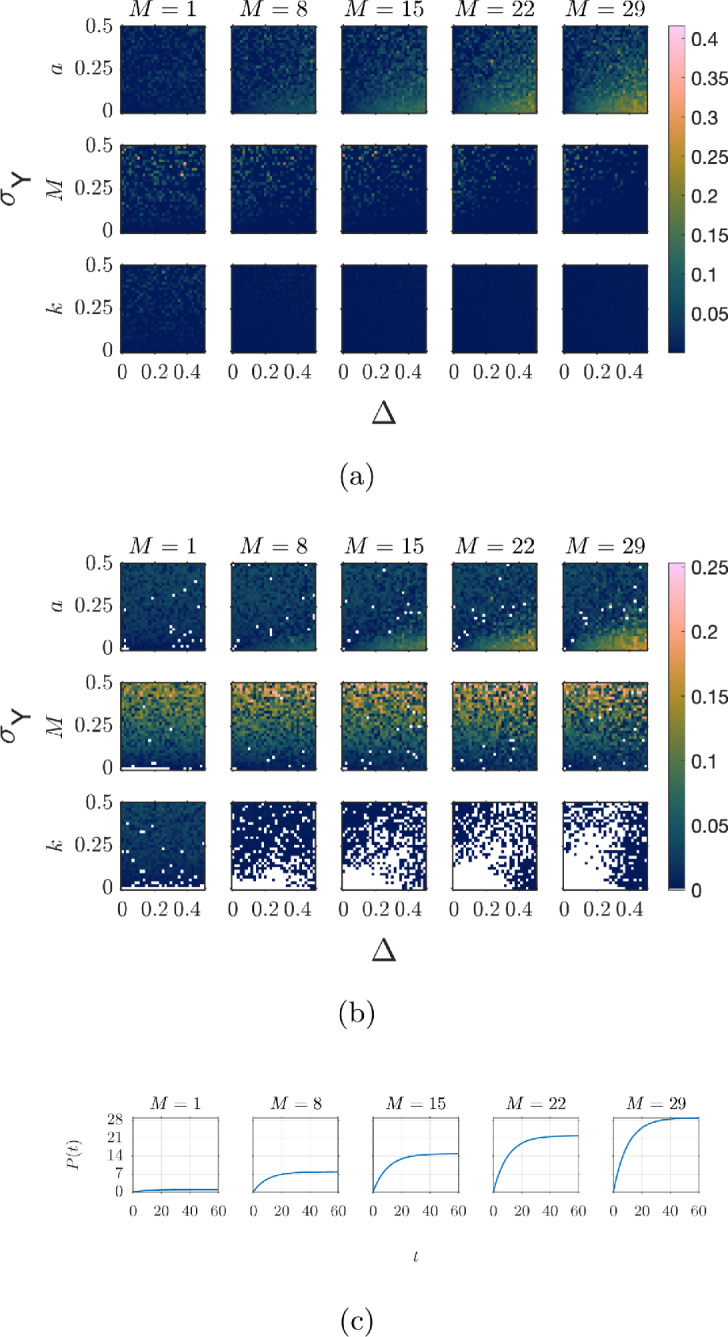
Bayesian parameter estimation metrics with the carrying capacity, *M*, varied across each column and the carrying capacity and start point set at $k=0.1$, and $P(0)=0.1$ respectively. Rows correspond to metrics for each parameter. Each heatmap shows the metric calculated from 8 samples at each time-point with varying $\Delta$ and $\sigma _Y$ values in the generated data. Figures indicate (a) the absolute error in the MAP estimate; **(b)** the distance from the true parameter value of the 95% credibility interval, white indicates the true parameter value is contained within the credibility interval; (c) shows the model output for the given set of parameters

**Fig. 22:**
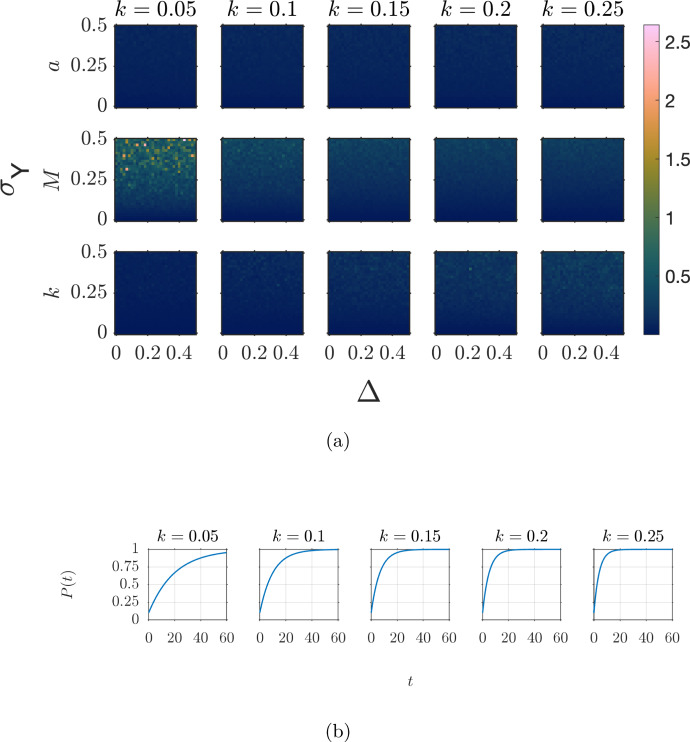
**(a)** The width of the 95% credibility interval with the rate parameter, *k*, varied across each column and the carrying capacity and start point set at $M=1$, and $a=0.1$ respectively. Each row corresponds to the CI of a different inference parameter. Each heatmap shows the width of the CI from 8 samples at each time-point with varying $\Delta$ and $\sigma _Y$ values in the generated data. **(b)** shows the model output for the given set of parameters

**Fig. 23:**
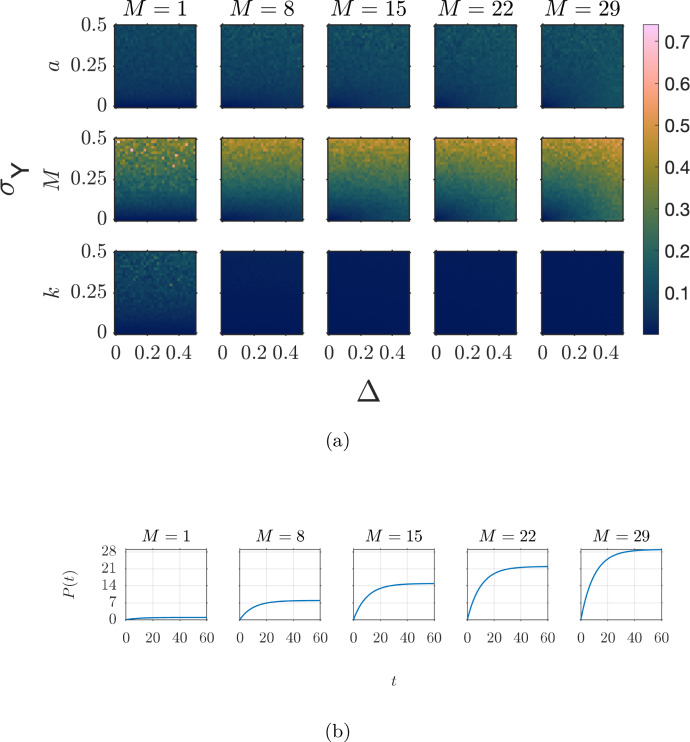
**(a)** The width of the 95% credibility interval with the rate parameter, *k*, varied across each column and the carrying capacity and start point set at $M=1$, and $a=0.1$ respectively. Each row corresponds to the CI of a different inference parameter. Each heatmap shows the width of the CI from 8 samples at each time-point with varying $\Delta$ and $\sigma _Y$ values in the generated data. **(b)** shows the model output for the given set of parameters

**Fig. 24:**
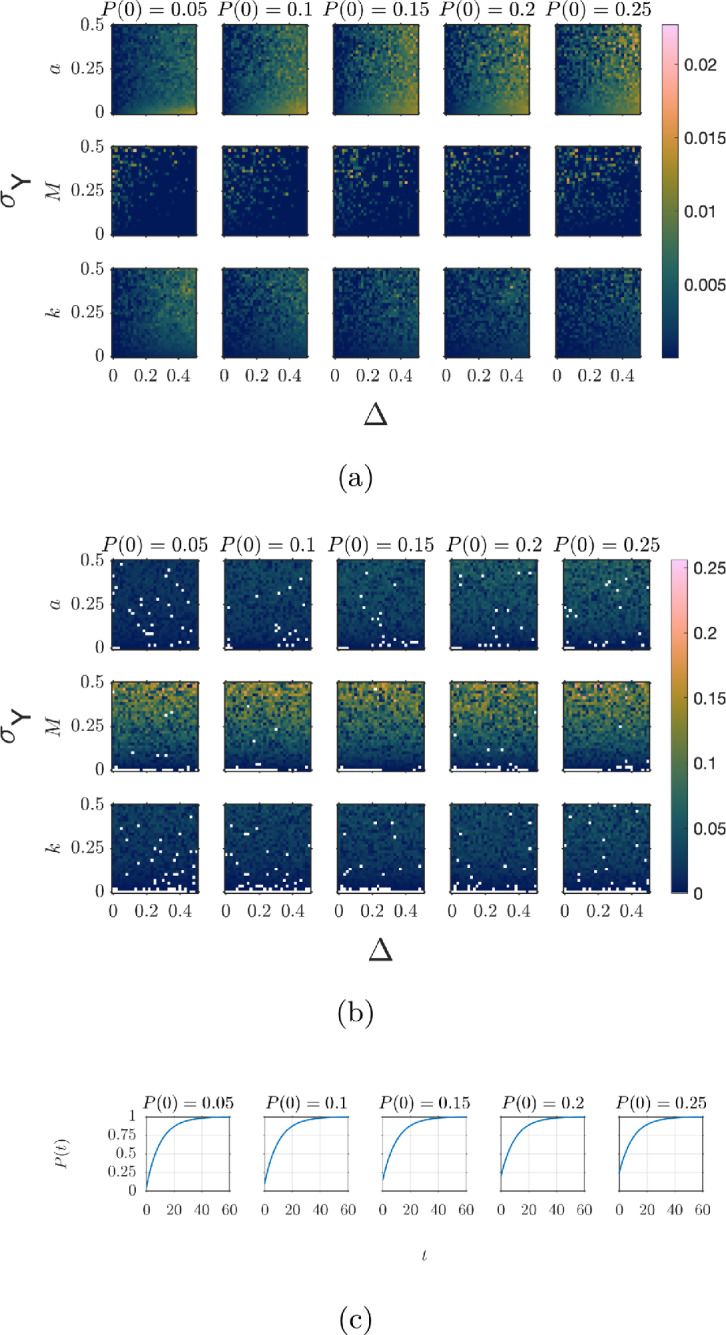
Bayesian parameter estimation metrics with the initial condition, *P*(0), varied across each column and the carrying capacity and start point set at $M=1$, and $k=0.1$ respectively. Rows correspond to metrics for each parameter. Each heatmap shows the metric calculated from 8 samples at each time-point with varying $\Delta$ and $\sigma _Y$ values in the generated data. Figures indicate **(a)** the absolute error in the MAP estimate; **(b)** the distance from the true parameter value of the 95% credibility interval, white indicates the true parameter value is contained within the credibility interval; **(c)** shows the model output for the given set of parameters

**Fig. 25:**
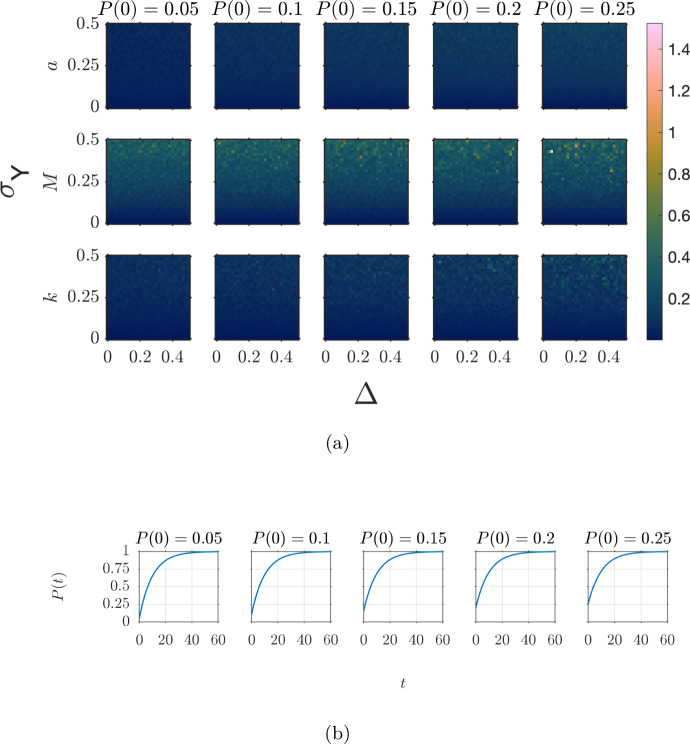
**(a)** The width of the 95% credibility interval with the initial condition, *P*(0), varied across each column and the carrying capacity and start point set at $M=1$, and $k=0.1$ respectively. Each row corresponds to the CI of a different inference parameter. Each heatmap shows the width of the CI from 8 samples at each time-point with varying $\Delta$ and $\sigma _Y$ values in the generated data. (b) shows the model output for the given set of parameters

**Fig. 26:**
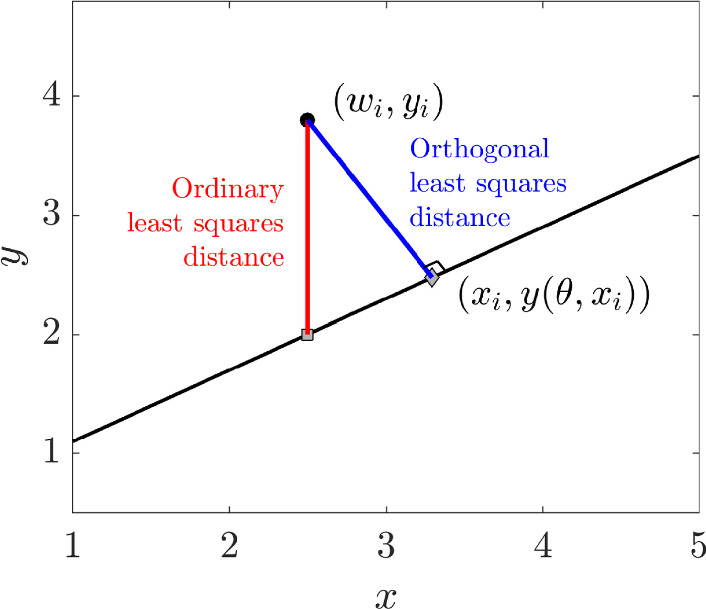
Illustration of the difference between ordinary least squares and orthogonal least squares

**Fig. 27:**
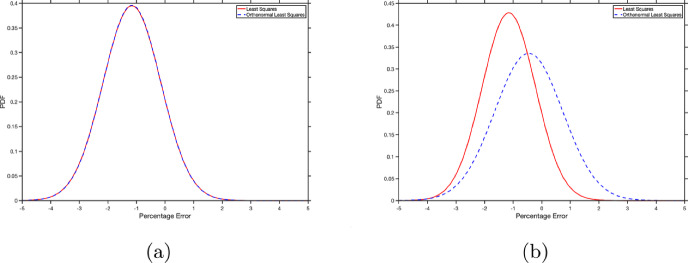
The density of the relative error (%) of the rate parameter *k* from inference of 100 generates datasets with (a) $\theta = [0.1,0.1,1]$ with $\sigma = \frac{1}{60}$, and (b) $\theta = [6,0.1,60]$ with $\sigma = 1$. Each generated dataset consisted of 8 samples at each time point

## Data Availability

The code used to produce and fit the data in this study is available on GitHub (https://github.com/brocksherlockmaths/Does-Timing-Matter).

## SMC Implementation and Metrics

For the models in this study, all of the model parameters, $\theta$, are non-negative. So, exponential priors, with mean 0.5, are used throughout the study. Posterior distributions are approximated using a sequential Monte Carlo (SMC) method (see (Chopin 2002; Del Moral et al. 2006) for SMC methods). The particular implementation used in this study is based on the work in Bon et al. (2021); South et al. (2019). All SMC implementations are performed with $N=1000$ particles, effective sample size (ESS) resampling threshold of *N*/2, and a probability of all particles moving at least once during Markov chain Monte Carlo (MCMC) of 0.99.

***Metrics used to Assess Inference*** To assess the quality of inference from Bayesian methods, we introduce a number of metrics. These metrics are gathered from the posterior distributions; we use the maximum a posteriori (MAP) estimate, and the credibility interval (both width of interval, whether the true parameter value is contained in the interval, and how far the true parameter value is from being in the interval when it is not contained). These metrics are illustrated in Fig. 15.

The MAP estimate is analogous to the maximum likelihood estimate (MLE) with a frequentist approach. The MAP is given by

22 $$\begin{aligned} \bar{\theta }_{MAP} = \arg \,\!\max _{\theta } \pi (\theta \vert y). \end{aligned}$$

That is, the MAP is the mode of the posterior distribution.

We define the bounds of the 95% credibility interval (*CI*) for each parameter as the 2.5-percentile, $CI_{2.5}$, and the 97.5-percentile, $CI_{97.5}$, of the marginal posteriors. This gives the interval width as $CI_{97.5} - CI_{2.5}$.

For the *i*th parameter, $\theta _i$, we define a distance from the true parameter value to the CI as

23 $$\begin{aligned} CI_{dist} = {\left\{ \begin{array}{ll} 0, & \text {if }\theta _i \in [CI^i_{2.5}, CI^i_{97.5}] \\ \min \{\vert \theta _i - CI^i_{97.5}\vert , \vert \theta _i - CI^i_{2.5}\vert \}, & \text {otherwise}, \end{array}\right. } \end{aligned}$$

where $\theta _i$ is the true parameter value and $CI^i$ refers to the corresponding credibility interval.

## Additional Plots for GLUT4 Case Study

Figures 16, 17, and 19 show the width of the 95% credibility interval for varying values of *k*, *M*, and *a* respectively.

Figures 11b, 12b, and 18b show the distance of the true parameter value to the 95% credibility interval, with red indicating the true value is within the interval, for varying values of *k*, *M*, and *a* respectively.

***Small Sample Sizes*** Figures 20, 21, 22, 23, 24 and 25 replicate those described in Section 3.2.2 but with 8 replicates per time point, as opposed to the 1000 used in the section. The same trends are evident for both sample sizes. The smaller sample size results only in less clear trends as the sampling has a greater effect on the metrics.

## Illustration of Orthogonal Regression with Scaled Data

Orthogonal least squares, sometimes referred to as *total least squares*, is a method to account for errors in a continuous independent variable; see Equation (19). While ordinary least squares considers the ‘vertical distance’ between a model output and a data point, orthogonal least squares considers the shortest distance between the model output and the data point. See Figure 26 for an illustration of the two least squares methods.

Using Equation (17) with $\eta =1$, we assume that *P*(0) and *M* are known (so that only *k* is inferred) and investigate the errors in estimates of *k* using ordinary least squares and orthonormal least squares. Datasets were generated using a normally distributed error in the equation. That is, data is

$$\begin{aligned} Y= (P(0)-M)e^{-kx} + M + \epsilon , \end{aligned}$$

where $\epsilon$ is normally distributed. The set of parameters is $\theta = (P(0),k,M)$.

We consider two *true* parameter sets: $\theta = [0.1,0.1,1]$ with $\sigma _\epsilon = \frac{1}{60}$, and $\theta = [6,0.1,60]$ with $\sigma _\epsilon = 1$. For each parameter set, 100 datasets were generated with 8 samples at each time point. We consider a biased measurement error with the true time of measurement $X=W+U$, where errors *U* are uniformly distributed. That is, $U \sim \mathcal {U}(0,1/6)$, a delay on time measurements of up to 10 seconds. The rate parameter, *k*, was then inferred for each dataset and the relative error calculated. The probability density for the relative error is shown in Figure 27.

It can be seen that the orthogonal least squares does not consistently correct for the error in time. For the case where $M=1$, the orthogonal least squares is close to the ordinary least squares estimate. However, when $M=60$ the orthogonal least squares gives better (less biased) estimates. This may be due to the scale of the vertical axis compared to the horizontal. Considering the data, measurements are taken over 60 minutes, so the horizontal axis runs from zero to 60. The orthogonal least squares is able to correct for the error better when the vertical axis is of a similar scale, i.e., the data runs between zero and 60. It seems that when the data is normalised so that the vertical scale is much smaller, the shortest distance between a data point and the model, is approximately the vertical distance. This is problematic for biological experiments, such as TIRF microscopy data, as these datasets are often normalised to maximum observed fluorescence levels, setting the highest data point around one.

## Example of Likelihood Construction

In this section, we construct the likelihood functions for classical error and Berkson error under the assumption of normally distributed error terms. Normally distributed errors are a common assumption and hence chosen as an example here.

We begin with the usual likelihood,

24 $$\begin{aligned} \phi _{Y\vert X}(y; y(\theta , x)) = \frac{1}{\sqrt{2 \pi \sigma _\epsilon ^2}}\exp \left( -\frac{(y-y(\theta ,x))^2}{2\sigma _\epsilon ^2}\right) , \end{aligned}$$

where we assume data has its mean given by the model output $y(\theta , x)$ with variance $\sigma _\epsilon ^2$.

We assume for both the classical $(W=X+U)$ and Berkson $(X=W+U)$ error cases that $U\sim \mathcal {N}(0,\sigma _u)$. So, for the classical error the observations *W* are normally distributed around the true value *X*, giving

25 $$\begin{aligned} \phi _{W\vert X}(w,x) = \frac{1}{\sqrt{2 \pi \sigma _u^2}}\exp \left( -\frac{(w-x)^2}{2\sigma _u^2}\right) . \end{aligned}$$

For the Berkson error case, the true value *X* is normally distributed about the observed *W*. This gives,

26 $$\begin{aligned} \phi _{X\vert W}(x,w) = \frac{1}{\sqrt{2 \pi \sigma _u^2}}\exp \left( -\frac{(x-w)^2}{2\sigma _u^2}\right) . \end{aligned}$$

For the classical error case we also need to prescribe a distribution for the true values *X*. Here, we assume they are normally distributed (as is the case for the data generated in Figure 1a) such that $X\sim (\mu _x, \sigma _x^2)$. This give

27 $$\begin{aligned} \phi _{X}(x) = \frac{1}{\sqrt{2 \pi \sigma _x^2}}\exp \left( -\frac{(x-\mu _x)^2}{2\sigma _x^2}\right) . \end{aligned}$$

Now, assume there are *N* pairs of observed data $(w_i,y_i)$ for $i=1,\dots ,N$. The likelihood for the classical error case is found by evaluating Equation (20) for each of the *N* pairs of data and taking the product. Substituting Equations (24), (25), and (27) into Equation (20) and taking the product gives the likelihood

$$\begin{aligned} \begin{aligned} L(\theta \vert y)&= \prod _{i=1}^N\int _{-\infty }^{\infty } \frac{1}{\sqrt{2\pi \sigma _\epsilon ^2}} \exp \!\left( -\frac{(y_i - y(\theta ,x))^2}{2\sigma _\epsilon ^2}\right) \\&\qquad \qquad \frac{1}{\sqrt{2\pi \sigma _u^2}} \exp \!\left( -\frac{(w_i - x)^2}{2\sigma _u^2}\right) \frac{1}{\sqrt{2\pi \sigma _x^2}} \exp \!\left( -\frac{(x - \mu _x)^2}{2\sigma _x^2}\right) \, dx \\&= \prod _{i=1}^N \frac{1}{\sqrt{8\pi ^3 \sigma _\epsilon ^2 \sigma _u^2 \sigma _x^2}} \\&\qquad \qquad \int _{-\infty }^{\infty } \exp \!\left( -\frac{(y_i - y(\theta , x))^2}{2\sigma _\epsilon ^2} -\frac{(w_i - x)^2}{2\sigma _u^2} -\frac{(x - \mu _x)^2}{2\sigma _x^2} \right) \, dx. \end{aligned} \end{aligned}$$

Note that the integral is taken over $[-\infty ,\infty ]$ as this is the range of possible values for the true $x_i$ values under the assumptions of normal distributions. Taking the log-likelihood, gives

28 $$\begin{aligned} \ell (\theta \mid y)&= \sum _{i=1}^N \bigg \{ \ln \left[ \int _{-\infty }^{\infty } \exp \!\left( -\frac{(y_i - y(\theta ,x))^2}{2\sigma _\epsilon ^2} -\frac{(w_i - x)^2}{2\sigma _u^2} -\frac{(x - \mu _x)^2}{2\sigma _x^2} \right) \, dx \right] \nonumber \\&\qquad \qquad -\frac{1}{2}\ln \!\left( 8\pi ^3 \sigma _\epsilon ^2 \sigma _u^2 \sigma _x^2\right) \bigg \} \end{aligned}$$

Similarly, for the Berkson error case the likelihood is found by evaluating Equation (21) for each of the *N* pairs of data and taking the product. This differs from the classical error case in that we do not need to prescribe a distribution for the true values *X*, only the conditional distribution for *X* given *W*. Substituting Equations (24) and (26) into Equation (21) gives

$$\begin{aligned} L(\theta \mid y)&= \prod _{i=1}^N \int _x \frac{1}{\sqrt{2\pi \sigma _\epsilon ^2}} \exp \!\left( -\frac{(y_i - y(\theta ,x))^2}{2\sigma _\epsilon ^2} \right) \\&\qquad \qquad \frac{1}{\sqrt{2\pi \sigma _u^2}} \exp \!\left( -\frac{(x - w_i)^2}{2\sigma _u^2} \right) \, dx \\&= \prod _{i=1}^N \frac{1}{\sqrt{4\pi ^2 \sigma _\epsilon ^2 \sigma _u^2}} \int _{-\infty }^{\infty } \exp \!\left( -\frac{(y_i - y(\theta ,x))^2}{2\sigma _\epsilon ^2} -\frac{(x - w_i)^2}{2\sigma _u^2} \right) \, dx. \end{aligned}$$

This can again be written as the log-likelihood,

29 $$\begin{aligned} \ell (\theta \mid y)&= \sum _{i=1}^N \bigg \{ \ln \!\left[ \int _{-\infty }^{\infty } \exp \!\left( -\frac{(y_i - y(\theta ,x))^2}{2\sigma _\epsilon ^2} -\frac{(x - w_i)^2}{2\sigma _u^2} \right) \, dx \right] \nonumber \\&\qquad \qquad -\frac{1}{2}\ln \!\left( 4\pi ^2 \sigma _\epsilon ^2 \sigma _u^2\right) \bigg \}. \end{aligned}$$

Comparing Equations (28) and (29), it can be seen that there are differences in the terms due to the contribution of the distribution of the true *X* values $\phi _X$ under the assumption of classical errors.
